# Artificial Intelligence-Guided Prioritization and Experimental Evaluation of Synergistic Target Combinations for Breast Cancer Therapy

**DOI:** 10.3390/ph19091363

**Published:** 2026-08-28

**Authors:** Chunlai Feng, Qiuqi Feng, Shengnan She, Ruojing Yang, Mengru Li, Lu Gong, Mengjie Rui

**Affiliations:** 1 School of Pharmacy, Jiangsu University, Zhenjiang 212013, China; feng@ujs.edu.cn (C.F.); 2212315007@stmail.ujs.edu.cn (Q.F.); 23110700148@m.fudan.edu.cn (S.S.); 2222415036@stmail.ujs.edu.cn (R.Y.); 2212415016@stmail.ujs.edu.cn (M.L.); 2222515009@stmail.ujs.edu.cn (L.G.); 2 Research Center for Intelligent Healthcare and Digital Health Technology, Affiliated Yixing People’s Hospital of Jiangsu University, Yixing 214205, China; 3 China’s National Health Commission (NHC) Key Laboratory of Diagnosis and Therapy of Gastrointestinal Tumor, Gansu Provincial Hospital, Lanzhou 730000, China; 4 Key Laboratory of Zhenjiang, School of the Environment and Safety Engineering, Jiangsu University, Zhenjiang 212013, China; 5 Engineering and Technology Institute of Jiangsu University, Zhenjiang 212013, China

**Keywords:** artificial intelligence, breast cancer, target combination, inhibitor combination, drug synergy

## Abstract

**Background/Objectives:** Breast cancer exhibits substantial molecular heterogeneity, resulting in diverse therapeutic vulnerabilities and limiting the efficacy of single-agent therapies. Although multi-target strategies may offer improved therapeutic benefit, the systematic identification of synergistic higher-order target combinations remains challenging. This study aimed to identify and validate effective higher-order target combinations for heterogeneous breast cancer. **Methods:** DeepMDS, a previously developed deep learning-based multi-compound synergy prediction model, was used to prioritize candidate target combinations for breast cancer. Exhaustive two-target and three-target combinations were ranked in the gene-expression contexts of ER-positive luminal-like MCF-7 and triple-negative MDA-MB-231 breast cancer cells. The top-ranked target combinations were evaluated using single, pairwise, and triple small interfering RNA (siRNA) perturbations. Representative inhibitors were subsequently assessed in fixed-ratio combinations in MCF-7, MDA-MB-231, and 4T1 cells and in a 4T1 syngeneic mouse experiment. **Results:** BIRC5-NAMPT-TOP1 ranked first in both cell lines. Triple siRNA co-transfection targeting *BIRC5*, *NAMPT*, and *TOP1* produced the strongest antiproliferative effects, with inhibition rates of 62.94% in MCF-7 cells and 55.62% in MDA-MB-231 cells. The corresponding inhibitors, LQZ-7I, FK866, and topotecan, exhibited synergistic antiproliferative activity at multiple molar ratios, with combination index values below 1. The optimized 2.5:10:1 molar ratio showed strong synergy in MCF-7, MDA-MB-231, and 4T1 cells, with combination index values of 0.26, 0.19, and 0.15, respectively. In tumor-bearing mice model, the triple-inhibitor regimen achieved a tumor inhibition rate of 62.05%. Topotecan-containing groups showed hematological alterations, whereas no statistically detectable elevations were observed in the measured terminal serum hepatic or renal biomarkers. **Conclusions:** The BIRC5-NAMPT-TOP1 combination showed reproducible phenotypic activity in the tested genetic and pharmacological models. These findings support the use of DeepMDS as a hypothesis-generation tool for higher-order target prioritization.

## 1. Introduction

Breast cancer is the cancer with the highest incidence worldwide and the most common cause of cancer death in women [1,2]. In addition to surgery, radiotherapy, chemotherapy, and endocrine therapy, molecular targeted therapy has significantly improved the clinical management of breast cancer owing to its high specificity and relatively low toxicity [3,4]. Nevertheless, durable disease control remains difficult to achieve in many cases, and treatment failure caused by limited efficacy, tumor recurrence, and acquired resistance continues to pose a major clinical challenge [5,6].

One major reason for this unsatisfactory outcome is the pronounced molecular heterogeneity of breast cancer. Breast cancer comprises several molecular subtypes, including Luminal A, Luminal B, human epidermal growth factor receptor 2 (HER2)-enriched, and Basal-like, which differ substantially in prognosis, therapeutic response, and biological vulnerability [7,8]. Owing to this heterogeneity, therapeutic responses vary markedly across tumors, and monotherapies targeting a single gene or pathway are frequently inadequate for durable disease control [9,10,11]. By contrast, combination therapy that concurrently modulates multiple oncogenic processes may provide greater therapeutic benefit and reduce the likelihood of resistance [12]. Therefore, the development of rational multi-target therapeutic strategies is of considerable importance for improving outcomes in heterogeneous breast cancer.

Identification of synergistic therapeutic combinations remains challenging because conventional experimental screening approaches are time-consuming and resource-intensive [13]. In recent years, computational approaches, particularly machine learning and deep learning methods, have emerged as efficient tools for predicting synergistic antitumor combinations because of their high-throughput capability, reproducibility, and ability to integrate complex multidimensional biological data [14,15,16,17,18]. For instance, Jeon et al. [19] developed a synergy prediction model based on extremely randomized trees, which incorporated genomic profiles of tumor cell lines, drug characteristics, and target information to predict pairwise drug synergies in specific cancer types. Liu and Xie [20] proposed TranSynergy, a mechanistically guided deep learning model with a self-attention architecture, which effectively combined gene dependency profiles, gene-gene interactions, and drug-target data to predict drug synergy.

Despite these advances, most existing studies have focused primarily on pairwise drug interactions and have not systematically explored higher-order combinations. More importantly, because the synergistic effects of drug combinations ultimately arise from coordinated perturbation of molecular targets and their associated signaling networks, identifying synergistic target combinations is more mechanistically meaningful than directly screening drug combinations. To address this limitation, we applied DeepMDS, a previously developed deep learning-based model that integrates a 215-gene expression profile from tumor cell lines, a 1093-element multi-hot target representation, and drug response data to predict pseudo-IC_50_ values as a relative measure of the synergistic antitumor potential of candidate target combinations [21].

In this study, we applied DeepMDS to systematically identify higher-order target combinations for breast cancer under two heterogeneous molecular contexts, including MCF-7 cells and MDA-MB-231 cells. MCF-7 represents an estrogen receptor-positive, luminal-like epithelial context, whereas MDA-MB-231 represents a triple-negative, basal-B/claudin-low, mesenchymal-like context [22,23,24]. Because cell-line-specific gene-expression profiles constitute part of the DeepMDS input, using the same cell lines for subsequent genetic and pharmacological experiments enabled context-matched evaluation of the model-prioritized combinations. The comparison was designed to determine whether a candidate combination retained activity across divergent molecular backgrounds or showed a stronger context preference, rather than to establish efficacy across all breast cancer subtypes. Top-ranked target combinations and lower-ranked comparator combinations were selected for experimental assessment in these two cell lines, and target perturbation was evaluated using single, pairwise, and triple siRNA transfections. The top-ranked target set was then translated to a fixed-ratio small-molecule combination and evaluated in vitro and in a 4T1 syngeneic mouse model. Through this workflow, we aimed to establish a data-driven strategy for discovering multi-target therapeutic combinations for heterogeneous breast cancer.

## 2. Results

### 2.1. Prediction of Synergistic Target Combinations Using the DeepMDS

After integration and deduplication of the collected datasets, 1093 unique tumor-related targets were retained. Exhaustive enumeration of unordered target sets without target repetition generated 596,778 two-target combinations and 217,028,266 three-target combinations, yielding a total of 217,625,044 candidate target sets.

Each candidate target set was encoded as a 1093-dimensional binary multi-hot vector, which was concatenated with the 215-dimensional gene-expression representation of either MCF-7 or MDA-MB-231 cells to form a 1308-dimensional input vector. For each cell-line expression context, candidate target vectors were generated and evaluated in computational batches. Thus, 435,250,088 prediction instances were evaluated across the two cell-line contexts; however, a single dense matrix of 435,250,088 × 1308 was neither constructed nor retained in memory.

The DeepMDS output was used as a relative pseudo-IC_50_ prioritization score. Lower scores indicated higher model-based prioritization. These values should not be interpreted as experimentally measured IC_50_ values, calibrated probabilities of synergy, or formal estimates of prediction uncertainty.

Among all candidate target sets, BIRC5-NAMPT-TOP1 produced the lowest pseudo-IC_50_ scores in both the MCF-7 and MDA-MB-231 expression contexts, with scores of −13.23 and −15.09, respectively, and ranked first in both contexts (Table 1). Its stable top ranking across the two tested expression backgrounds led us to select BIRC5-NAMPT-TOP1 as the lead candidate for subsequent experimental evaluation.

For rank-contrast comparison, the target combination represented experimentally as HSPA1A/HSPA1B (HSP72)-NAMPT-MTOR was selected because it showed a more context-dependent ranking, ranking 850th in MCF-7 cells and 37th in MDA-MB-231 cells. The BIRC5-NAMPT-TOP1 lead candidate and the HSP72-NAMPT-MTOR rank-contrast comparator were therefore advanced for genetic evaluation. Because only a limited number of target combinations were experimentally examined, these ranking results represent candidate prioritization rather than comprehensive validation of model performance across the entire target-combination space.

### 2.2. Effect of siRNA Co-Transfection on mRNA Expression of Target Gene

To verify target suppression after either single-siRNA transfection or simultaneous transfection of the three siRNAs corresponding to each candidate combination, reverse-transcription quantitative polymerase chain reaction (RT-qPCR) analysis was conducted in both MCF-7 and MDA-MB-231 cells. Cells were transfected with either single or combined siRNAs targeting *BIRC5*, *NAMPT*, and *TOP1*, or *HSP72*, *NAMPT*, and *MTOR*, and mRNA levels were measured. For the HSP72 arm, *HSPA1A* and *HSPA1B* encode highly similar, stress-inducible HSP70 proteins that are commonly grouped under the designation HSP72. Therefore, the *HSP72* siRNA in this study was designed to suppress both *HSPA1A* and *HSPA1B* together.

**Figure 1:**
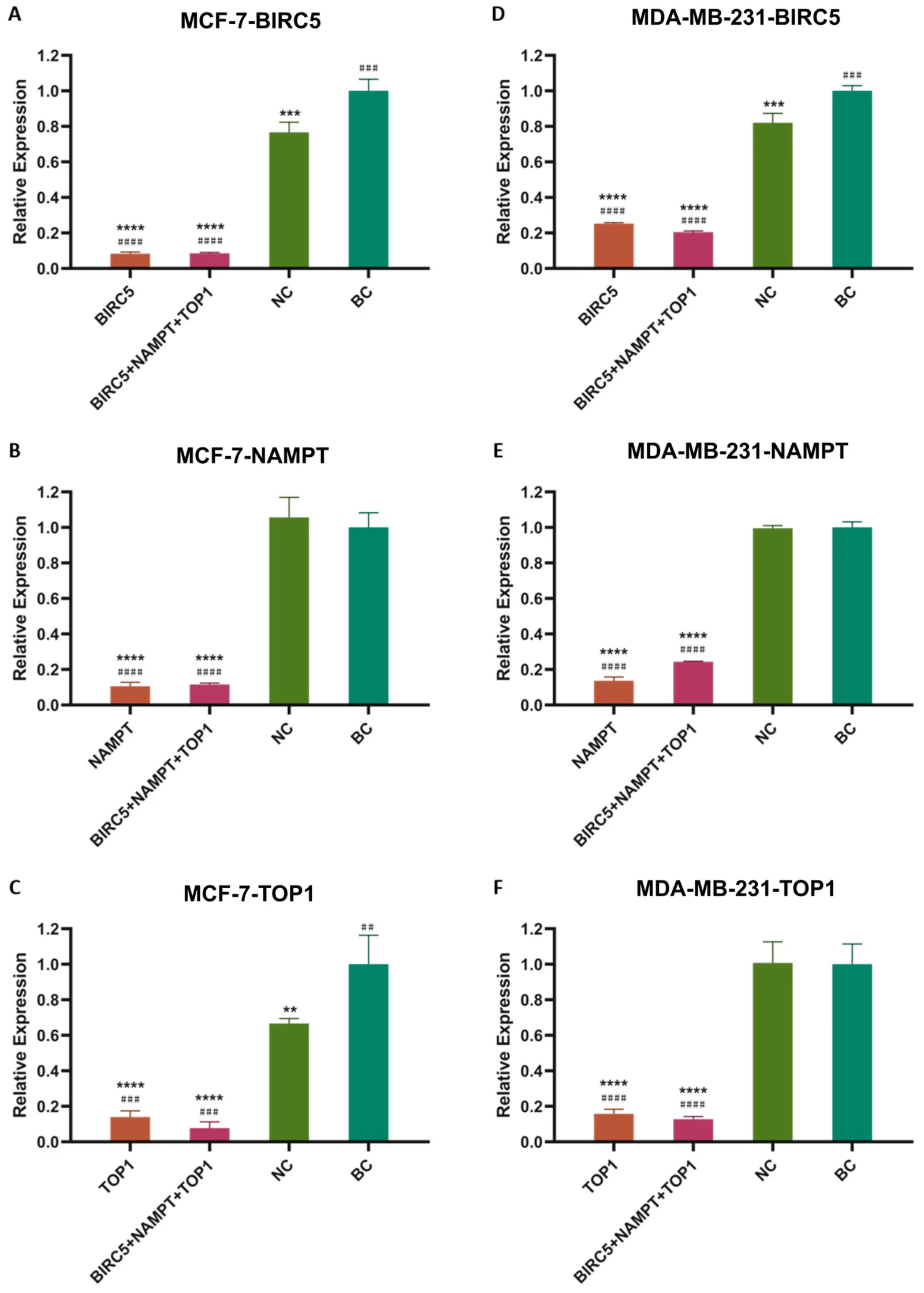
Relative mRNA expression levels of *BIRC5*, *NAMPT*, and *TOP1* in MCF-7 (**A**–**C**) and MDA-MB-231 (**D**–**F**) cells after single and co-transfection of siRNAs. Significance: ** *p* < 0.01, *** *p* < 0.001, **** *p* < 0.0001 versus blank control (BC); ## *p* < 0.01, ### *p* < 0.001, #### *p* < 0.0001 versus negative control (NC). Data are presented as the mean ± SD (*n* = 3).

In MCF-7 cells, single siRNA transfection resulted in knockdown efficiencies of 91.62% for *BIRC5*, 88.42% for *NAMPT*, and 86.02% for *TOP1*, while triple co-transfection achieved knockdown rates of 91.48%, 88.40%, and 92.23%, respectively (Figure 1A–C). Similarly, in MDA-MB-231 cells, the reductions after single-target transfection were 74.71%, 86.40%, and 84.29%, respectively, and those after triple co-transfection were 79.50%, 75.63%, and 87.21% (Figure 1D–F). These findings indicated that single siRNA transfection of *BIRC5*, *NAMPT*, and *TOP1* was effective, and that co-transfection of siRNAs efficiently silenced all three target genes simultaneously in both cell lines.

**Figure 2:**
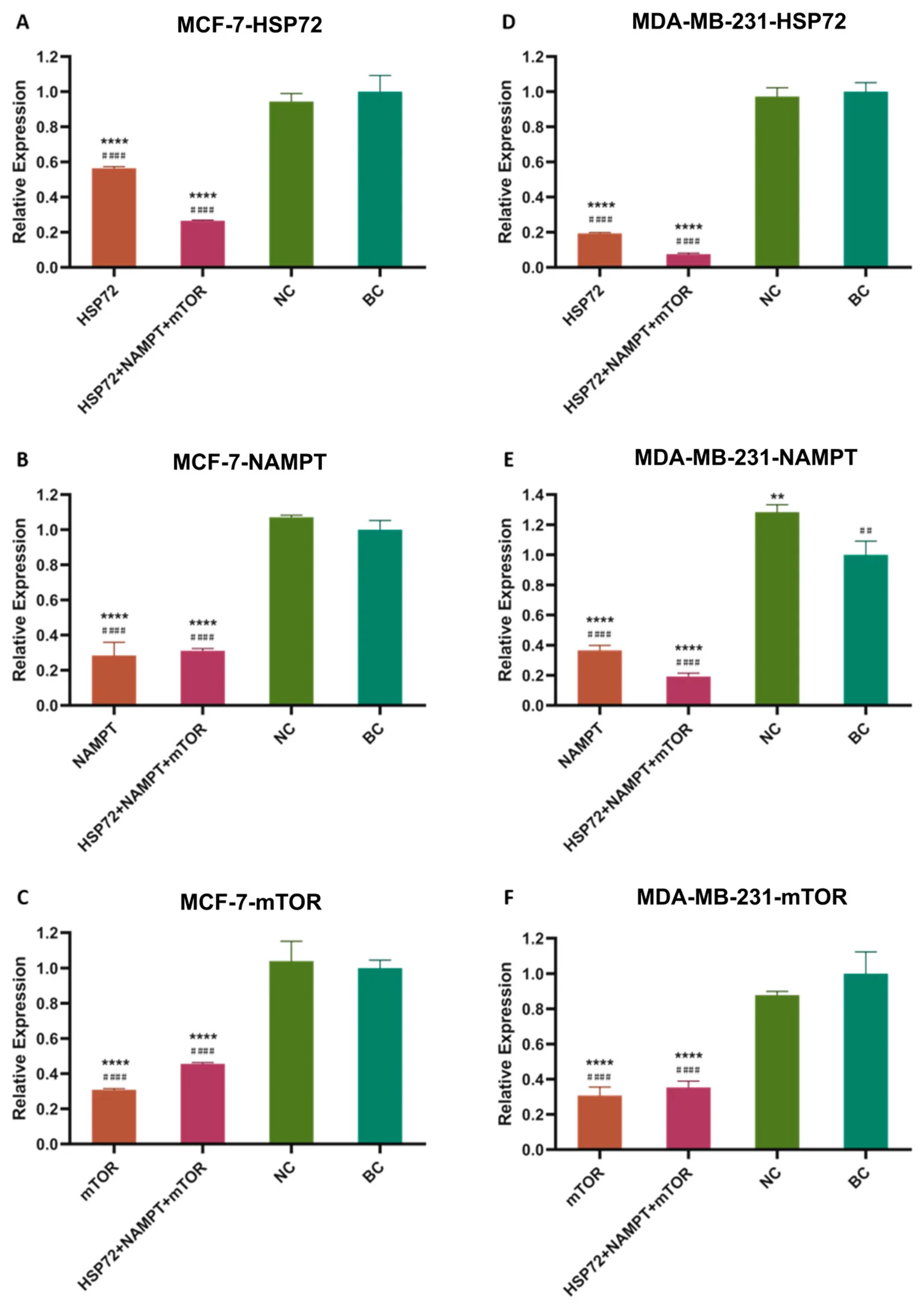
Relative mRNA expression levels of *HSP72* (*HSPA1A*/*HSPA1B*), *NAMPT*, and *MTOR* in MCF-7 (**A**–**C**) and MDA-MB-231 (**D**–**F**) cells after single-siRNA or triple-siRNA transfection. Significance: ** *p* < 0.01, **** *p* < 0.0001 versus BC; ## *p* < 0.01, #### *p* < 0.0001 versus NC. Data are presented as the mean ± SD (*n* = 3).

For the comparator combination, siRNAs targeting *HSP72*, *NAMPT*, and *MTOR* significantly downregulated mRNA expression in both single and triple transfection groups (Figure 2). In MCF-7 cells, transfection with the *HSPA1A*/*HSPA1B*-targeting siRNA reduced the combined *HSPA1A*/*HSPA1B* transcript abundance by 43.52% (Figure 2A), whereas triple co-transfection with the *HSPA1A*/*HSPA1B*-, *NAMPT*-, and *MTOR*-targeting siRNAs resulted in a 73.41% reduction. For *NAMPT* and *MTOR*, single-siRNA transfection produced reductions of 71.56% and 69.17%, respectively, whereas triple-siRNA co-transfection produced reductions of 68.81% and 54.34%, respectively (Figure 2B,C).

In MDA-MB-231 cells, the combined *HSPA1A*/*HSPA1B* transcript abundance was reduced by 80.62% after single-siRNA transfection and by 92.47% after triple-siRNA co-transfection (Figure 2D). For *NAMPT* and *MTOR*, single-siRNA transfection produced reductions of 63.40% and 69.26%, respectively, whereas triple-siRNA co-transfection produced reductions of 80.84% and 64.62%, respectively (Figure 2E,F).

Collectively, these results show that the individual siRNAs reduced their corresponding transcript signals and that triple-siRNA co-transfection enabled simultaneous suppression of all three targets in both cell lines.

### 2.3. Effects of Single, Pairwise, and Triple siRNA-Mediated Target Silencing on Cell Viability

To determine the individual and combined contributions of the predicted targets, CCK-8 assays were performed after transfection with each individual siRNA, all three pairwise-siRNA combinations, or the corresponding triple-siRNA combination in MCF-7 and MDA-MB-231 cells. The single-siRNA groups were used to assess the contribution of each target, whereas the pairwise- and triple-siRNA groups were used to determine whether simultaneous perturbation of multiple targets produced a greater reduction in cell viability.

**Figure 3:**
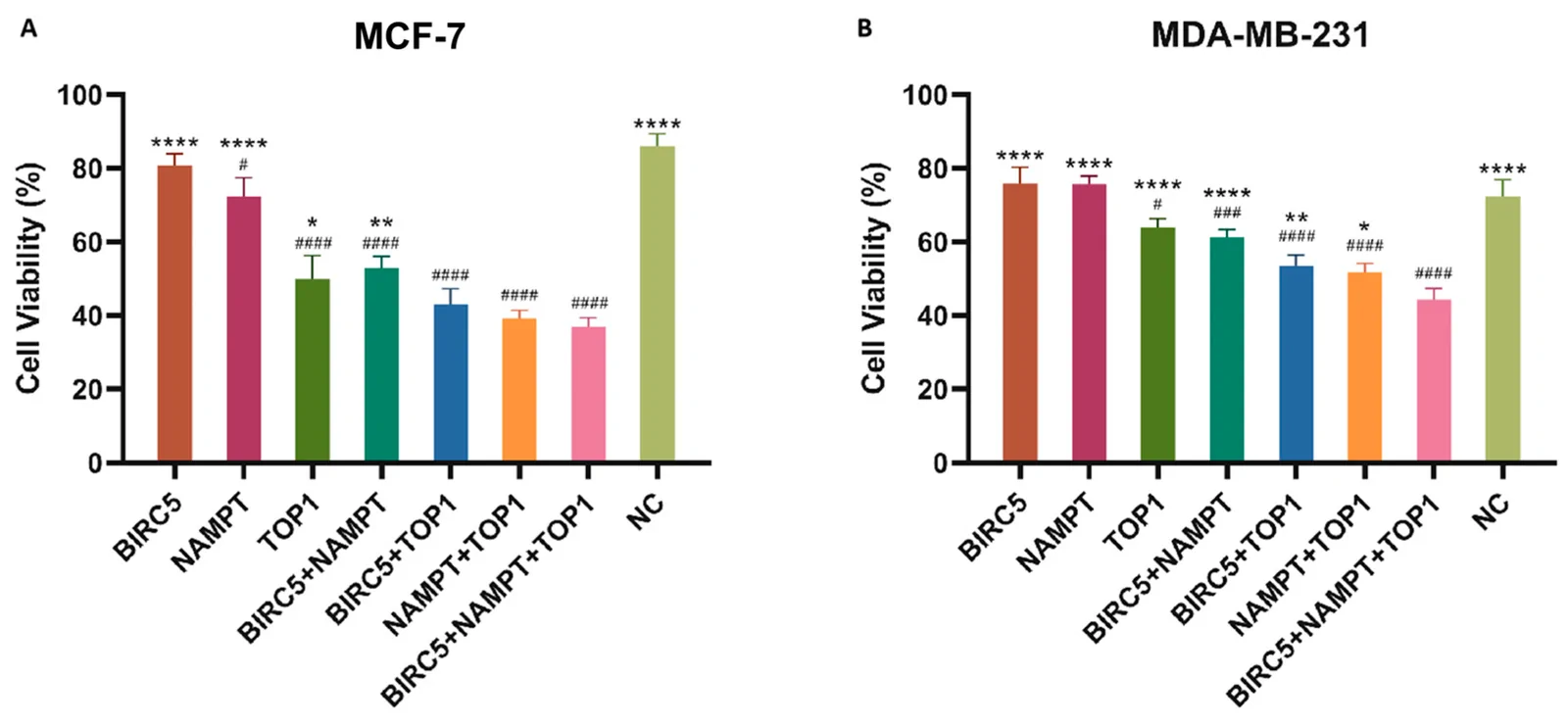
The cell viability of MCF-7 (**A**) and MDA-MB-231 (**B**) cells after single, pairwise, or triple transfection with siRNAs targeting *BIRC5*, *NAMPT*, and *TOP1*. Data are presented as the mean ± SD (*n* = 5). Statistical significance was evaluated using one-way ANOVA followed by Tukey’s multiple-comparison test. Significance: compared with NC group, # *p* < 0.05, ### *p* < 0.001, #### *p* < 0.0001; comparison with the siRNA co-transfection group of *BIRC5*, *NAMPT*, and *TOP1*, * *p* < 0.05, ** *p* < 0.01, **** *p* < 0.0001.

For the top-ranked BIRC5-NAMPT-TOP1 combination, distinct antitumor effects were observed in both cell lines (Figure 3). In MCF-7 cells, single transfection with *BIRC5* siRNA did not significantly reduce cell viability compared to the negative control (NC) group. However, transfection with *NAMPT* or *TOP1* siRNAs, as well as co-transfection of any two or all three siRNAs, significantly suppressed proliferation (Figure 3A). The most pronounced inhibition was observed in the triple co-transfection group with an inhibition rate of 62.94%, which was significantly stronger than all single-target groups. In MDA-MB-231 cells, transfection with *TOP1* siRNA, any two-gene combinations, and especially the triple-target siRNA co-transfection all significantly inhibited proliferation compared to the negative control (NC) group (Figure 3B). The strongest inhibition was again observed in the triple-target group with an inhibition rate of 55.62%, showing statistical significance relative to all other groups. These results indicate that co-silencing *BIRC5*, *NAMPT*, and *TOP1* produced the greatest reduction in cell viability among the tested single-, pairwise-, and triple-siRNA conditions.

**Figure 4:**
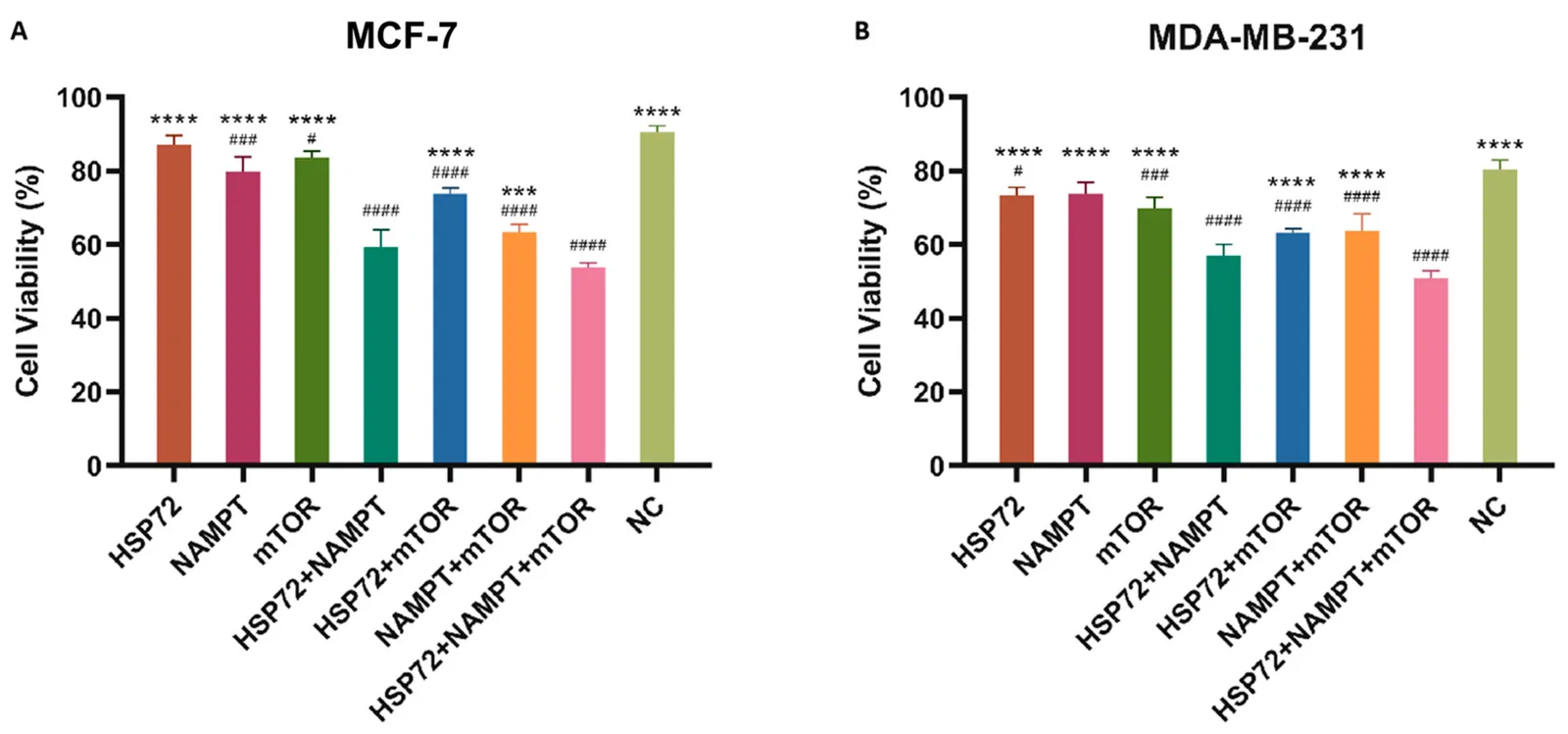
The cell viability of MCF-7 (**A**) and MDA-MB-231 (**B**) cells after single, pairwise, or triple transfection with siRNAs targeting *HSPA1A*/*HSPA1B* (*HSP72*), *NAMPT* and *MTOR*. Data are presented as the mean ± SD (*n* = 5). Statistical significance was evaluated using one-way ANOVA followed by Tukey’s multiple-comparison test. Significance: compared with NC group, # *p* < 0.05, ### *p* < 0.001, #### *p* < 0.0001; comparison with *HSP72*, *NAMPT*, *MTOR* siRNA co-transfection group, *** *p* < 0.001, **** *p* < 0.0001.

For the HSP72-NAMPT-MTOR combination, a similar but weaker pattern was observed. In MCF-7 cells, transfection with *NAMPT* or *MTOR* siRNAs, combinations of two siRNAs, and the triple-target group all significantly inhibited cell proliferation compared to the NC group (Figure 4A). The triple co-transfection yielded the strongest effect, yielding an inhibition rate of 46.22%, and was significantly more effective than any single siRNA treatment. In MDA-MB-231 cells, transfection with *HSP72* or *MTOR* siRNAs, as well as all combinations of two or three siRNAs, significantly inhibited proliferation (Figure 4B). The strongest inhibition occurred in the triple-target group with a value of 49.05%, significantly exceeding the effects of individual knockdowns.

Direct comparison of the two candidate combinations showed that co-silencing *BIRC5*, *NAMPT*, and *TOP1* produced stronger antiproliferative effects than co-silencing *HSP72*, *NAMPT*, and *MTOR* in both breast cancer cell lines. The greater phenotypic effect of BIRC5-NAMPT-TOP1 relative to the selected comparator was consistent with its higher model rank, although the present comparison does not establish the biological basis of the ranking or the model’s performance across the full target-combination space.

Overall, these findings aligned well with DeepMDS model predictions, supporting the ability of the DeepMDS-based ranking procedure to prioritize a target combination with a comparatively stronger functional effect in the two tested breast cancer cell lines. The BIRC5-NAMPT-TOP1 combination was selected for further investigation.

### 2.4. In Vitro Evaluation of the Corresponding Inhibitor Combinations

To pharmacologically validate the prioritized target combination, LQZ-7I, FK866, and topotecan were selected as representative inhibitors of BIRC5, NAMPT, and TOP1, respectively. The antiproliferative activities of these three compounds, used either alone or in combination, were subsequently evaluated in MCF-7, MDA-MB-231, and 4T1 cells.

**Figure 5:**
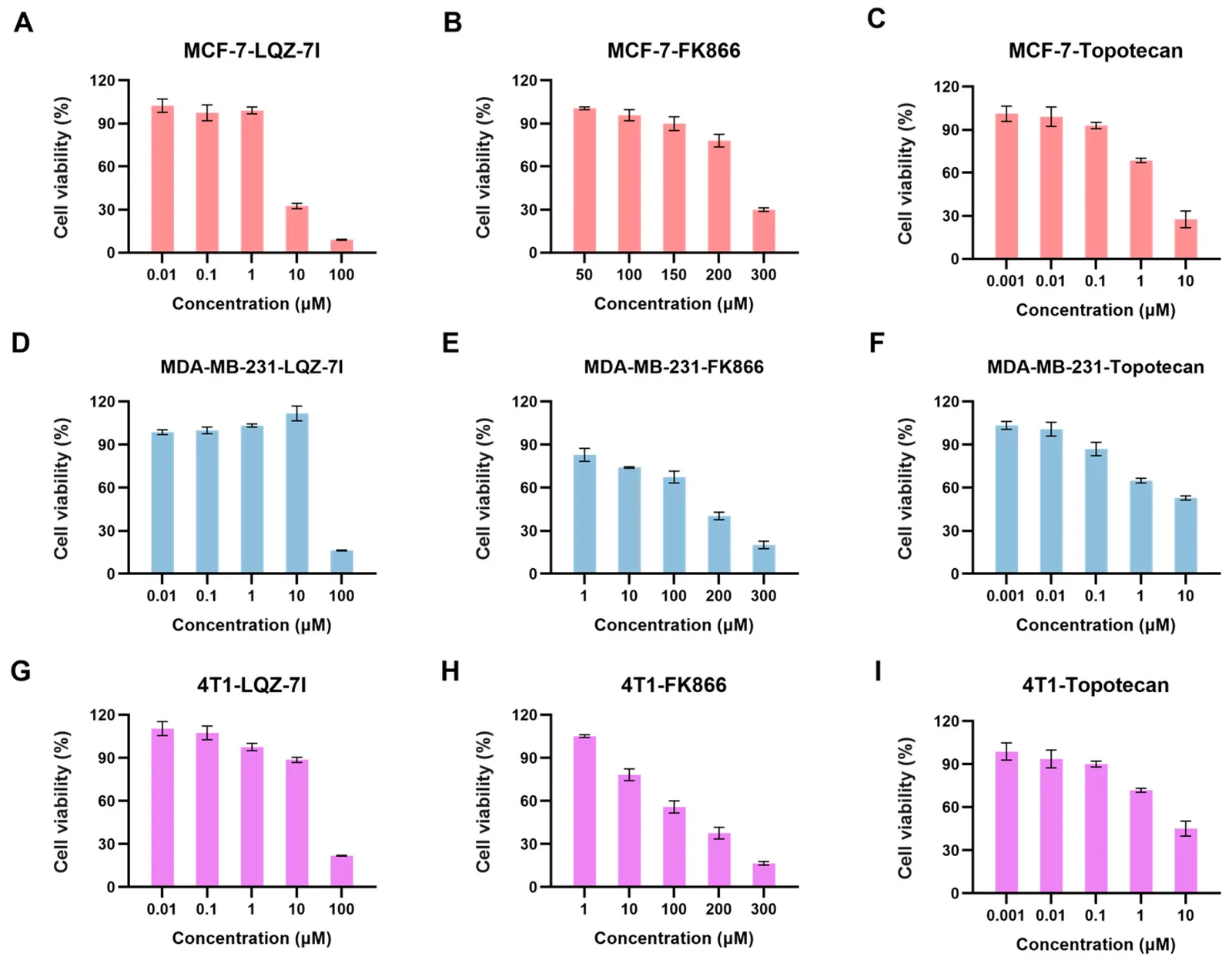
Effects of LQZ-7I, FK866 and topotecan on cell viability of MCF-7 (**A**–**C**), MDA-MB-231 (**D**–**F**), and 4T1 (**G**–**I**) cells. Data are presented as the mean ± SD (*n* = 4).

We first assessed the effects of the individual inhibitors on cell viability. In MCF-7 cells, LQZ-7I and topotecan showed potent inhibition with IC50 values of 6.75 μM and 2.80 μM, respectively, while FK866 demonstrated weak activity (IC50 = 253.50 μM) (Figure 5A–C). In MDA-MB-231 cells, topotecan was also the most active single agent, with an IC50 value of 9.09 μM, whereas LQZ-7I and FK866 showed moderate to weak inhibition (IC50 = 79.85 μM and 93.85 μM, respectively) (Figure 5D–F). The corresponding single-agent experiments were additionally performed in murine triple-negative breast cancer 4T1 cells. Topotecan achieved the lowest IC50 value of 6.76 μM, followed by LQZ-7I (41.38 μM) and FK866 (91.01 μM) (Figure 5G–I). The observed differences in the antiproliferative effects of LQZ-7I and FK866 among three cell lines suggest cell-line- and context-dependent pharmacological sensitivity, underscoring the necessity of developing more precise, subtype-tailored therapeutic strategies for breast cancer.

To eliminate inter-batch variability and more accurately assess drug synergy, we examined the antiproliferative effects of the three drugs individually and in four defined molar ratios: 2.5:10:1, 2.5:0.1:1, 10:10:1, and 10:0.1:1. At each ratio, the concentrations of all three components changed proportionally. The synergistic drug interaction was quantified by calculating combination index (CI) values at the 50% effect level.

**Figure 6:**
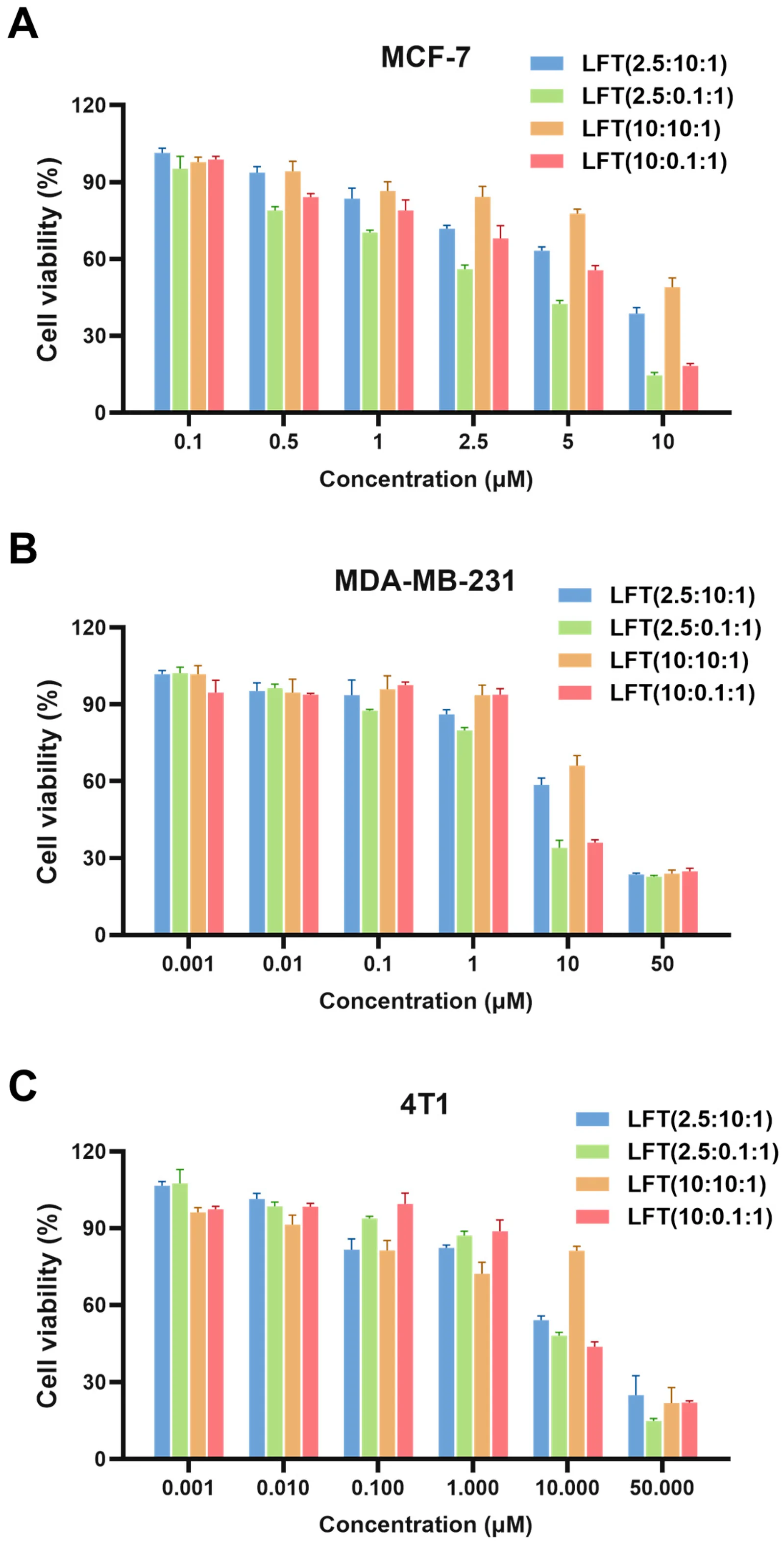
Fixed-ratio concentration-response profiles of LQZ-7I + FK866 + topotecan in MCF-7 (**A**), MDA-MB-231 (**B**), and 4T1 (**C**) cells. The x-axis represents the total nominal mixture concentration; the concentration of each component, including topotecan, changed proportionally according to the indicated molar ratio. Data are presented as the mean ± SD (*n* = 4).

In MCF-7 cells, all combinations demonstrated strong inhibitory effects and synergy, with IC_50_ values below 15 μM and CI values below 1.0 (Figure 6A; Table 2). Among them, the 2.5:10:1 ratio showed the strongest synergy (IC_50_ = 7.04 μM, CI = 0.26), while the 2.5:0.1:1 ratio had the highest inhibitory potency (IC_50_ = 2.74 μM, CI = 0.43). The weakest synergy was observed with the 10:10:1 ratio (IC_50_ = 11.46 μM, CI = 0.82), whereas the 10:0.1:1 ratio yielded an IC_50_ of 4.32 μM and a CI of 0.35.

A similar trend was observed in MDA-MB-231 cells. All combinations showed strong synergistic effects, with IC_50_ values below 20 μM and CI values below 0.5 (Figure 6B; Table 2). The 2.5:10:1 ratio again demonstrated the strongest synergy (IC_50_ = 13.16 μM, CI = 0.19), while the 2.5:0.1:1 ratio yielded the highest inhibitory potency (IC_50_ = 5.15 μM, CI = 0.21). Other ratios also exhibited synergy, for example, 10:10:1 with an IC_50_ value of 17.80 μM and a CI value of 0.23, and 10:0.1:1 with an IC_50_ value of 7.79 μM and a CI value of 0.37.

Furthermore, a ratio-dependent pattern on cell growth was also observed in 4T1 cells. All four fixed-ratio combinations demonstrated synergistic effects (Figure 6C; Table 2). The 2.5:10:1 ratio produced the lowest CI value (CI = 0.15; total-mixture IC_50_ = 10.35 μM), whereas the 2.5:0.1:1 ratio produced the lowest total-mixture IC_50_ value (8.57 μM; CI = 0.36). The 10:10:1 and 10:0.1:1 ratios produced total-mixture IC_50_ values of 23.05 and 8.77 μM and CI values of 0.42 and 0.65, respectively. These 4T1 data extend the pharmacological evaluation to the murine cell line used for tumor implantation and thereby improve continuity between the murine in vitro and in vivo experiments.

In summary, the triple-inhibitor combination of LQZ-7I, FK866, and topotecan exhibited consistent and strong synergistic antitumor effects across a range of molar ratios. The 2.5:10:1 molar ratio emerged as the most synergistic in three cell lines. Therefore, the 2.5:10:1 ratio was selected for subsequent efficacy evaluation in vivo.

### 2.5. In Vivo Evaluation of Anticancer Effects of Inhibitor Combinations

#### 2.5.1. Effects of Inhibitor Combinations on Tumor Growth in Tumor-Bearing Mice

**Figure 7:**
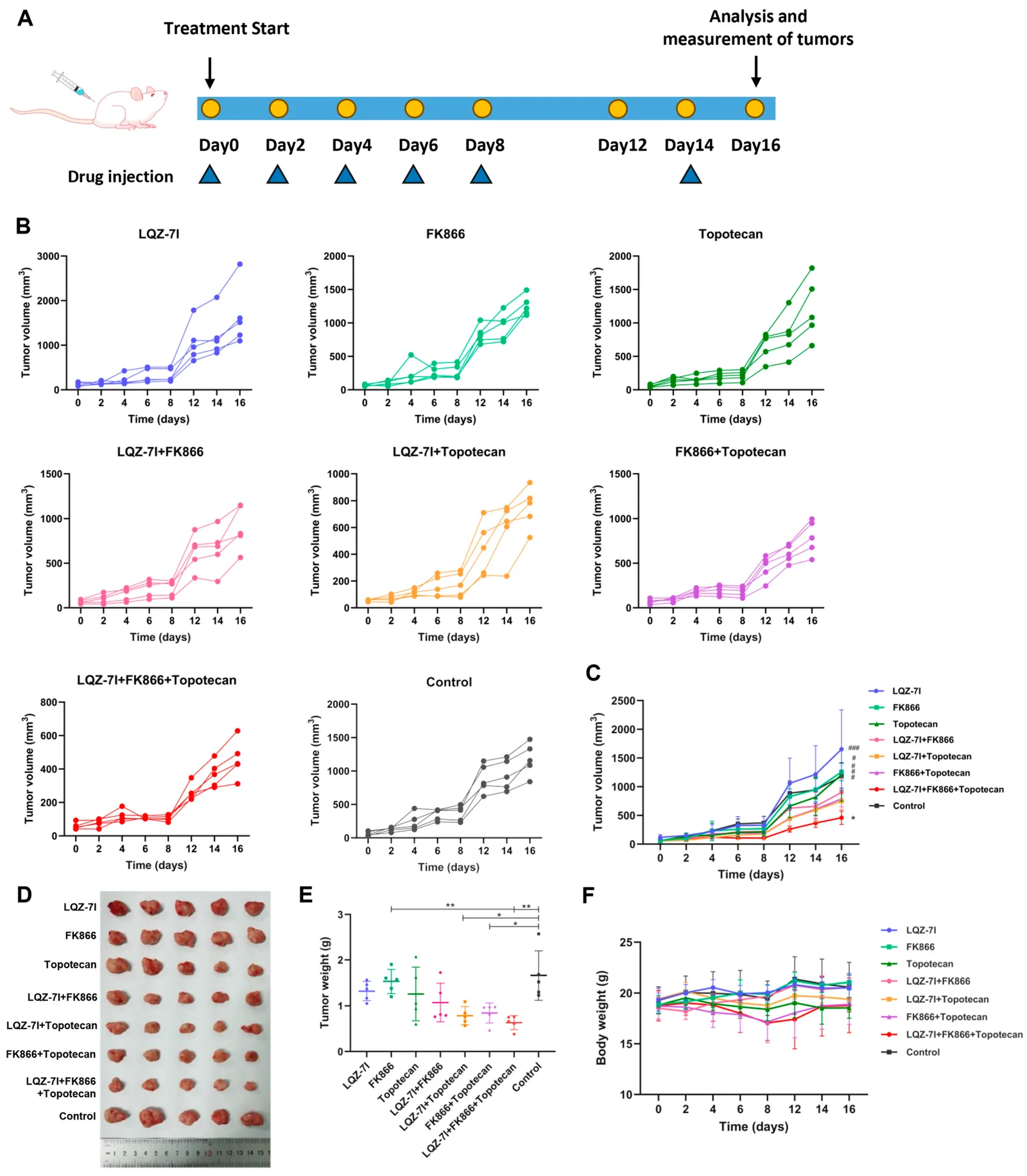
The in vivo antitumor effects of LQZ-7I, FK866, topotecan and their combinations in 4T1 tumor-bearing mice. (**A**) Schematic of drug administration, tumor volume monitoring, and analysis timeline. (**B**) Individual tumor volume growth curves following different treatments (*n* = 5 per group). (**C**) Average tumor volume growth curves following different treatments (*n* = 5 per group). (**D**) Representative images of excised tumors from sacrificed mice at the study end. (**E**) Terminal tumor weights at the study end. (**F**) Body weight changes during the treatment. Data are expressed as the mean ± standard deviation (SD; *n* = 5 per group). Significance: * *p* < 0.05, ** *p* < 0.01 vs. the control group; # *p* < 0.05, ### *p* < 0.001 vs. the LQZ-7I + FK866 + topotecan group.

All seven treatment groups and the vehicle-control group followed the same actual administration schedule (Figure 7A). Drugs or corresponding vehicle preparations were administered on Days 0, 2, 4, 6, 8, and 14. The final tumor-volume assessment and terminal sample collection were performed on Day 16.

Tumors in the control and single-agent treatment groups exhibited rapid and continuous growth. In contrast, tumor progression was markedly suppressed in all combination therapy groups. Notably, beginning on Day 6, the tumor volume in the LQZ-7I + FK866 + topotecan triple-drug group was significantly smaller than that of the control group. By Day 16, tumor volumes in the single-drug groups remained comparable to the control, whereas those in the triple-combination group were significantly reduced, demonstrating enhanced antitumor efficacy (Figure 7B,C).

The triple-inhibitor combination (LQZ-7I + FK866 + topotecan) exhibited the strongest tumor inhibition, achieving an inhibition rate of 62.05%. Dual-inhibitor combinations of LQZ-7I + topotecan and FK866 + topotecan showed moderate efficacy, with inhibition rates of 53.01% and 49.40%, respectively. The LQZ-7I + FK866 combination had a weaker effect, yielding an inhibition rate of 35.54%. In contrast, the monotherapies yielded limited inhibition: LQZ-7I (20.48%), FK866 (7.83%), and topotecan (24.10%) (Table 3).

Tumor weight measurements supported these findings. Significant reductions in tumor mass were observed in the triple-drug group and in both topotecan-containing dual-drug groups, compared with the control. No significant reductions were seen in the LQZ-7I + FK866 or monotherapy groups.

These results confirm that the combination of LQZ-7I, FK866, and topotecan exerted superior antitumor effects in vivo compared to monotherapies or dual-drug regimens.

#### 2.5.2. Effects of Inhibitor Combinations on Body Weight in Tumor-Bearing Mice

Drugs or corresponding vehicles were initially administered every 2 days on Days 0, 2, 4, 6, and 8. At the Day 8 assessment, mice in the FK866 + topotecan and LQZ-7I + FK866 + topotecan groups exhibited body-weight loss, reduced activity, and deterioration in general condition.

Following the Day 8 administration and welfare assessment, the dosing interval was extended from 2 to 6 days for all eight experimental groups, including the vehicle-control group. The next and final administration was therefore performed on Day 14, and the study endpoint was Day 16. Applying the modified schedule uniformly ensured that all groups remained aligned with respect to administration timing after Day 8.

Body-weight trajectories subsequently stabilized during the remaining observation period (Figure 7F). Nevertheless, because the schedule was modified in response to treatment-associated welfare findings, tumor-growth, body-weight, hematological, and serum-biochemistry observations obtained after Day 8 reflect the schedule-adapted regimen. Stabilization of body weight after the modification does not establish tolerability of the originally planned every-other-day regimen.

#### 2.5.3. Effects of LQZ-7I, FK866 and Topotecan on Liver and Kidney Function in Tumor-Bearing Mice

To evaluate the potential hepatic and renal toxicity of LQZ-7I, FK866, and topotecan that have been administered either individually or in combination, we measured key liver and kidney function biomarkers in serum from tumor-bearing mice. Terminal blood samples for serum biochemistry analysis were collected on Day 16, two days after the final administration on Day 14.

**Figure 8:**
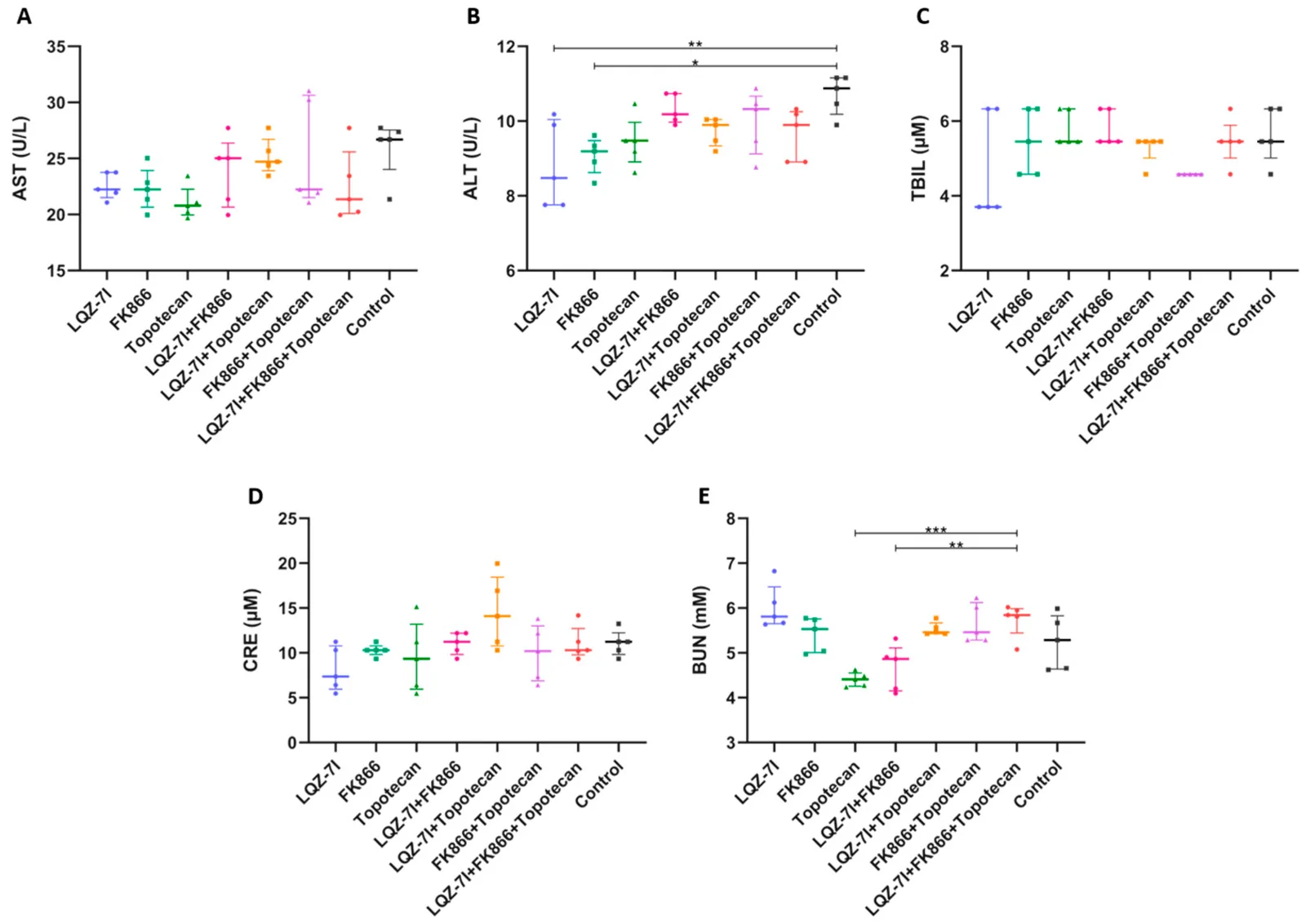
Effects of LQZ-7I, FK866, topotecan and their combinations on liver and kidney function indexes in 4T1 tumor-bearing mice. (**A**) AST; (**B**) ALT; (**C**) TBIL; (**D**) CRE; and (**E**) BUN. Each symbol represents one mouse, and the horizontal lines and error bars indicate the mean ± SD (*n* = 5 per group). Significance: * *p* < 0.05, ** *p* < 0.01, *** *p* < 0.001.

Compared to the control group, monotherapy with LQZ-7I or FK866 significantly reduced serum alanine aminotransferase (ALT) activity, although the levels remained within the normal physiological range. In contrast, no significant changes in ALT were observed in any of the combination treatment groups. For other markers of hepatic and renal function, including aspartate aminotransferase (AST), total bilirubin (TBIL), creatinine (CRE), and blood urea nitrogen (BUN), no statistically significant differences were detected between treatment groups and the control (Figure 8). These results imply that the hepatic or renal toxicity of therapeutic groups was limited.

No statistically detectable treatment-associated increase was observed in the measured serum hepatic and renal biomarkers under the modified dosing schedule. These results suggest that LQZ-7I, FK866, and topotecan, whether used alone or in combination, do not induce significant liver or kidney toxicity under the tested dosing regimen.

#### 2.5.4. Effects of Inhibitor Combination on Hematological Parameters in Tumor-Bearing Mice

Complete blood counts were obtained from terminal blood samples collected on Day 16, two days after the final administration. Topotecan is associated with severe hematological toxicity, particularly neutropenia and leukopenia [25]. To characterize treatment-associated hematological changes in topotecan-based regimens, we evaluated complete blood counts (CBC) in tumor-bearing mice treated with LQZ-7I, FK866, or topotecan, either as monotherapies or in combination.

**Figure 9:**
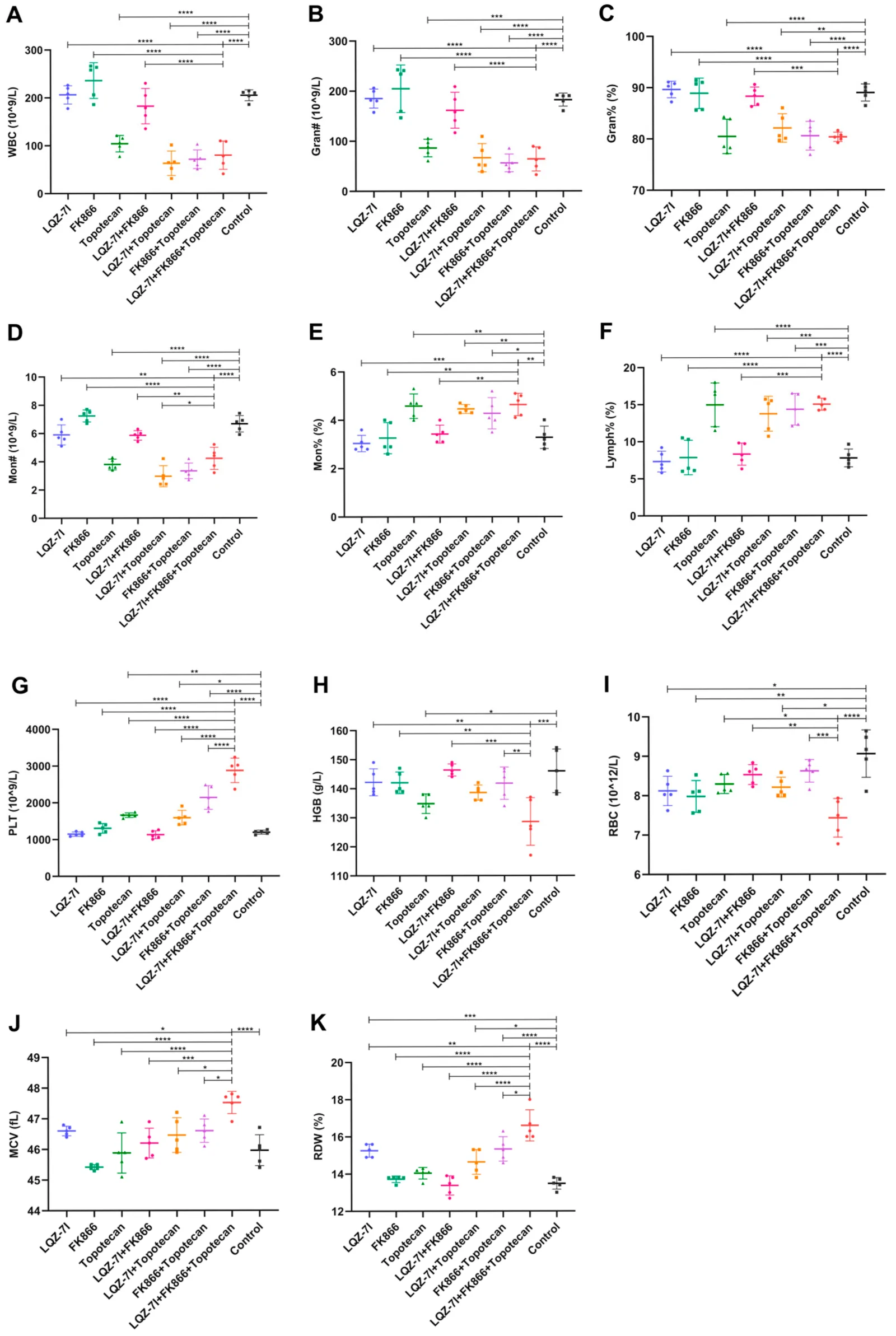
Effects of LQZ-7I, FK866, topotecan and their combinations on hematological parameters of tumor-bearing mice. (**A**) White blood cell (WBC) count. (**B**) Granulocyte count (Gran#). (**C**) Granulocyte percentage (Gran%). (**D**) Monocyte count (Mon#). (**E**) Monocyte percentage (Mon%). (**F**) Lymphocyte percentage (Lymph%). (**G**) Platelet count (PLT). (**H**) Hemoglobin (HGB) level. (**I**) Red blood cell (RBC) count. (**J**) Mean corpuscular volume (MCV). (**K**) Red blood cell distribution width (RDW). Each symbol represents one mouse, and the horizontal lines and error bars indicate the mean ± SD (*n* = 5 per group). Significance: * *p* < 0.05, ** *p* < 0.01, *** *p* < 0.001, **** *p* < 0.0001 vs. control group.

Compared to the control group, the four treatment groups containing topotecan (topotecan alone, LQZ-7I + topotecan, FK866 + topotecan, and LQZ-7I + FK866 + topotecan) significantly reduced white blood cell (WBC), neutrophil, and monocyte counts, as well as neutrophil percentage. Conversely, these treatments led to significant increases in monocyte percentage, lymphocyte percentage, and platelet count (Figure 9A–G). Topotecan-containing regimens produced detectable hematological alterations (Figure 9A–K). Under the modified dosing schedule, the triple-combination group did not show a statistically detectable further reduction in WBC or neutrophil counts relative to topotecan alone in this small cohort. The absence of statistical significance does not demonstrate equivalent toxicity, non-inferiority, or mitigation of topotecan-associated hematotoxicity. In addition, no significant alterations were observed in lymphocyte count, hematocrit (HCT), mean corpuscular hemoglobin concentration (MCHC), mean corpuscular hemoglobin (MCH), mean platelet volume (MPV), or platelet distribution width (PDW) in any treatment group compared to the control.

Collectively, topotecan-containing regimens produced detectable hematological alterations under the tested conditions. Under the modified dosing schedule, the triple-combination group did not show a statistically detectable further reduction in WBC or neutrophil counts relative to the topotecan-monotherapy group in this small cohort. However, the absence of a statistically significant difference does not demonstrate equivalent hematological toxicity, non-inferiority, or mitigation of topotecan-associated hematotoxicity.

## 3. Discussion

The principal contribution of this study is the establishment of a target-first computational-experimental workflow for prioritizing and evaluating higher-order therapeutic combinations. Using our previously developed DeepMDS model, all eligible two-target and three-target combinations within a curated set of 1093 tumor-related targets were ranked independently in the gene-expression contexts of MCF-7 and MDA-MB-231 cells. The BIRC5-NAMPT-TOP1 combination received the top rank in both contexts and was subsequently evaluated through single, pairwise, and triple siRNA perturbations, followed by fixed-ratio pharmacological testing of representative inhibitors. The selected inhibitor combination was further examined in 4T1 cells and in a syngeneic mouse model. This progression from target-space prioritization to genetic and pharmacological evaluation represents the distinguishing feature of the present work.

The present application differs from the earlier drug-combination use of DeepMDS by decoupling candidate generation from a predefined compound library. Rather than beginning with available drugs, the current workflow first enumerated idealized higher-order target combinations and subsequently translated the prioritized target combination into a pharmacological implementation. This target-first strategy expands the searchable space beyond combinations already represented by specific compounds and provides a rational route for experimental triage of otherwise intractable candidate numbers. At the same time, the DeepMDS output should be interpreted as a relative prioritization score rather than a measured IC_50_, a calibrated probability of synergy, or evidence that adjacent ranks are statistically distinguishable. The model was originally trained using pharmacological-response labels, and its application to idealized target combinations relies on the transferability of the underlying target-vector representation. The current results therefore demonstrate the feasibility of this workflow for candidate generation and prioritization, rather than general validation of DeepMDS for arbitrary genetic target combinations.

The use of MCF-7 and MDA-MB-231 cells provided two deliberately contrasting human breast cancer contexts. MCF-7 represents an estrogen receptor-positive, luminal-like epithelial background, whereas MDA-MB-231 represents a triple-negative, basal-B/claudin-low and mesenchymal-like background. Because the cell-line-specific expression profile constitutes part of the DeepMDS input, the same target combination can receive different scores and ranks depending on the molecular context. Notably, BIRC5-NAMPT-TOP1 ranked first in both cell lines, whereas the comparator represented experimentally as HSPA1A/HSPA1B (HSP72)-NAMPT-MTOR ranked 850th in MCF-7 cells and 37th in MDA-MB-231 cells. This design enabled comparison of a candidate with stable prioritization across the two contexts against a candidate with a more context-dependent ranking.

The ShinyTHOR analysis further contextualized this cell-line selection. Descriptive visualization of BIRC5, NAMPT, TOP1, HSPA1A, HSPA1B, and MTOR across breast-derived cancer cell lines showed substantial inter-cell-line variation in their basal expression patterns (Supplementary Figure S2) [26]. MCF-7 and MDA-MB-231 occupied different positions in the six-gene hierarchical clustering, and neither represented a uniformly high- or low-expression state for all investigated targets. Their selection was therefore based on the correspondence between the DeepMDS input contexts and their contrasting biological backgrounds rather than on expression extremes. Basal transcript abundance alone does not define target dependency, protein activity, or drug sensitivity, but the observed heterogeneity illustrates why context-matched evaluation is important when prioritizing multi-target interventions. It also supports the future inclusion of broader panels spanning ER-positive luminal, HER2-positive, basal-like, claudin-low/triple-negative, and patient-derived breast cancer systems.

The siRNA experiments provided an initial functional assessment of the model-prioritized target combinations. Evaluation of the complete single-, pairwise-, and triple-siRNA conditions showed that simultaneous targeting of BIRC5, NAMPT, and TOP1 produced the greatest reduction in cell viability among the tested conditions in both MCF-7 and MDA-MB-231 cells. This target set also produced a greater phenotypic effect than the selected HSPA1A/HSPA1B-NAMPT-MTOR comparator. Within this limited two-candidate comparison, the direction of the functional difference was consistent with the relative DeepMDS rankings. Nevertheless, testing one top-ranked and one lower-ranked target set is insufficient to establish a general rank–response relationship or to explain why BIRC5-NAMPT-TOP1 received the top model score. Prospective testing of multiple high-, intermediate-, and low-ranked combinations will be required to evaluate rank stability and the broader predictive value of the prioritization procedure.

The gene silencing result should also be interpreted according to the endpoints measured. The result of CCK-8 assay reflects cellular metabolic activity and viability rather than a formal genetic-interaction model. The greater effect of triple knockdown is therefore best described as an enhanced combinatorial phenotype rather than definitive genetic synergy. In addition, target suppression was confirmed at the transcript level, whereas survivin/BIRC5, NAMPT, and TOP1 protein abundance and functional target engagement were not measured. Because transcript and protein responses may differ in magnitude and kinetics, the present data link multiplex transcript perturbation with reduced viability but do not establish complete protein depletion or phenotype-protein causality. Time-resolved protein quantification, direct target-engagement measurements, and siRNA-resistant rescue experiments would provide a more complete evaluation of this relationship [27,28].

The biological functions of BIRC5, NAMPT, and TOP1 nevertheless provide a coherent hypothesis for the observed combinatorial phenotype. NAMPT supports the NAD^+^ salvage pathway and thereby contributes to metabolic maintenance and NAD^+^-dependent stress responses. TOP1 regulates DNA topology, and its inhibition can generate replication-associated and DNA-damage stress. Survivin/BIRC5 contributes to mitotic progression and cellular resistance to apoptotic and genotoxic stress [29,30,31,32]. Concurrent perturbation of these processes could therefore reduce metabolic and repair capacity while simultaneously lowering the threshold for loss of viability. This hypothesis provides biological plausibility for prioritizing the triad, but it remains a working model. Intracellular NAD^+^ abundance, PARP activity, PARylation, DNA-damage signaling, apoptosis, cell-cycle distribution, and metabolic responses were not directly measured. Establishing the interaction mechanism will require matched single-, pairwise-, and triple-treatment experiments combined with time-resolved biochemical measurements and pathway-specific rescue analyses.

Translation of the prioritized target set into a fixed-ratio pharmacological combination provided a second and orthogonal level of evaluation. LQZ-7I, FK866, and topotecan were used as representative agents associated with survivin/BIRC5, NAMPT, and TOP1 inhibition, respectively [29,33,34,35]. Across MCF-7, MDA-MB-231, and 4T1 cells, all four tested fixed ratios produced CI values below 1. The 2.5:10:1 ratio consistently produced the lowest CI value, whereas the 2.5:0.1:1 ratio produced the lowest IC_50_ value. This distinction is informative: the ratio showing the strongest calculated interaction was not necessarily the ratio with the greatest apparent potency. The results therefore emphasize the importance of composition when evaluating higher-order combinations and support systematic ratio optimization in future studies using design-of-experiments or response-surface approaches [36]. The four tested ratios sampled only a limited region of the compositional space and require further global optimization of the combination composition and dosing schedule.

The pharmacological data also define important constraints on interpretation. LQZ-7I remains an experimental survivin-directed compound, whereas FK866 has demonstrated dose-limiting toxicities during clinical evaluation. In the present 48 h cell viability experiment, FK866 showed unusually high apparent IC_50_ values compared with the substantially lower concentrations at which NAMPT inhibition and NAD^+^ depletion have been reported elsewhere. Differences in exposure duration, cellular background, culture conditions, and assay endpoint may have contributed to this discrepancy. However, because NAD^+^ depletion, direct NAMPT target engagement, and metabolite rescue were not measured, the loss of viability at the highest FK866 concentrations cannot be attributed exclusively to NAMPT inhibition, and concentration-dependent off-target effects cannot be excluded. The CI values are considered as empirical pharmacological interaction measures under the tested conditions rather than definitive evidence of NAMPT-specific molecular synergy. Similarly, the fractional amount of topotecan in a fixed-ratio combination describes combination composition but does not establish clinically meaningful dose sparing, reduced systemic exposure, or mitigation of topotecan toxicity.

The present study did not include head-to-head benchmarking against subtype-appropriate standard-of-care regimens. The experimental design focused on the internal evaluation of the model-prioritized triad by comparing the triple regimen with its constituent monotherapies and corresponding dual combinations, rather than on comparative therapeutic efficacy. Because clinically relevant reference regimens differ across hormone receptor-positive, HER2-positive, and triple-negative breast cancer, a single comparator evaluated in the 4T1 model would not provide a general benchmark for these distinct disease contexts. Future studies would therefore evaluate the prioritized target concept in human-derived, subtype-matched models using exposure-matched standard-of-care controls and prospectively fixed dosing schedules, together with pharmacokinetic and toxicological assessments.

The in vitro 4T1 experiments improved continuity between the murine cell-culture and tumor-implantation stages. The 2.5:10:1 ratio also produced the lowest CI value among the tested ratios in 4T1 cells, supporting evaluation of this composition in the corresponding syngeneic model. In the tumor-bearing mouse experiment, the triple regimen produced the lowest terminal tumor burden and the highest calculated tumor-inhibition rate among the tested groups. These results extend the pharmacological observation to one immunocompetent murine setting. However, 4T1 is not biologically equivalent to MCF-7 or MDA-MB-231 and was not an input context for the human DeepMDS rankings. The murine experiment therefore supports activity in the 4T1 setting but does not establish cross-species transferability of the ranking or efficacy in human luminal or triple-negative breast cancer. Moreover, because immune-cell composition and function were not examined, the study does not determine whether the intact host immune system contributed to the treatment response.

Interpretation of the animal findings is also shaped by the exploratory design. The group size of five mice per condition limited statistical precision and the characterization of inter-animal variability. In addition, the administration schedule was modified after body-weight loss, reduced activity, and deterioration in general condition were observed in the FK866 + topotecan and triple-combination groups. Consequently, the terminal tumor, body-weight, hematological, and biochemical findings reflect a schedule-adapted regimen rather than the originally planned every-other-day regimen. One mouse in the LQZ-7I group also exceeded the institutional 2000 mm^3^ tumor-volume criterion during the late observation period, representing an isolated humane-endpoint implementation deviation. These observations indicate that future efficacy studies need to use a prospectively established tolerable schedule, a statistically justified sample size, intensified late-stage tumor monitoring, and predefined stopping procedures.

The hematological and serum biochemistry findings should be viewed as treatment-associated laboratory observations rather than a comprehensive safety assessment. Topotecan-containing groups showed changes in several hematological parameters. The absence of a statistically detectable further reduction in WBC or neutrophil counts in the triple-combination group relative to topotecan alone does not establish equivalent toxicity or mitigation of topotecan-associated hematological effects. Likewise, the absence of treatment-associated elevations in the measured terminal serum hepatic and renal biomarkers does not exclude histological injury, cumulative toxicity, delayed toxicity, or effects in unexamined organs. Formal characterization of the therapeutic index will require prospective dose-escalation and maximum tolerated dose studies, pharmacokinetic and exposure-response analyses, longitudinal hematological monitoring, recovery cohorts, and histopathological examination of bone marrow, gastrointestinal tract, liver, kidney, spleen, and other relevant tissues.

The present study also defines several priorities for extending the computational framework. The current rankings are point estimates generated by a fixed model and do not include formal uncertainty intervals. No independent labeled dataset of higher-order genetic perturbations was available for external calibration, and only a small number of target combinations were evaluated experimentally. Therefore, future model assessment includes larger perturbational datasets, prospective testing across multiple rank strata, model ensembles or repeated training, rank-stability analyses, and comparison with target-compatible network and machine-learning baselines. Integration of cell-line-specific expression with genetic dependency, protein abundance, and context-specific interaction networks may further improve the biological relevance of target prioritization. Because the original training labels were pharmacological responses, systematic evaluation against controlled combinatorial genetic-perturbation data will be particularly important for defining the transferability of DeepMDS from drug-target representations to idealized target combinations.

In summary, this study demonstrates the feasibility of an end-to-end target-first workflow that connects exhaustive higher-order target prioritization with genetic perturbation, fixed-ratio pharmacology, and in vivo evaluation. The value of the approach lies not in introducing a new DeepMDS architecture or establishing a clinically optimized regimen, but in reducing a very large target-combination space to experimentally testable candidates while retaining cell-context information throughout the workflow. BIRC5-NAMPT-TOP1 emerged as a reproducibly prioritized candidate and showed the strongest phenotypic activity among the target combinations examined. These results support further investigation of this triad and provide a foundation for broader rank-response validation, mechanistic studies, and pharmacological optimization.

## 4. Materials and Methods

### 4.1. Materials

LQZ-7I (Cat. No. T8469) and FK866 (Cat. No. T2644) were purchased from TargetMol (Shanghai, China). Topotecan hydrochloride (Cat. No. T939252), polyoxyethylene castor oil (Cat. No. C804845), sodium sulfobutylether-β-cyclodextrin (Cat. No. C871854), and 1,2-propanediol (Cat. No. P815581) were obtained from Maclin (Shanghai, China). Dimethyl sulfoxide (DMSO; Cat. No. D2653) was purchased from Sigma (Shanghai, China), and absolute ethanol (Cat. No. 10009265) was obtained from Sinopharm Chemical Reagent Co., Ltd. (Shanghai, China). Dulbecco’s modified Eagle’s medium (DMEM, high glucose; Cat. No. 11965118), Roswell Park Memorial Institute-1640 medium (RPMI-1640; Cat. No. 11875119), and fetal bovine serum (FBS; Cat. No. 10091148) were purchased from Gibco (Thermo Fisher Scientific, Waltham, MA, USA). Penicillin-streptomycin solution (Cat. No. C0222) was obtained from Beyotime (Shanghai, China). Cell Counting Kit-8 (CCK-8; Cat. No. CK04) was obtained from DOJINDO Molecular Technologies (Kumamoto, Japan). siRNAs and GP-transfect-Mate transfection reagent were acquired from GenePharma (Suzhou, China). RNA-Quick Purification Kit (Cat. No. ES-RN001), Fast All-in-One RT Kit (Cat. No. ES-RT001), and qPCR Master Mix (Cat. No. ES-QP002) were provided by YiShan Biotech (Shanghai, China).

### 4.2. Prediction of Synergistic Target Combinations Using DeepMDS

To identify synergistic target combinations against breast cancer, tumor-related target information, including anti-tumor compound targets and known anti-tumor pathway molecules, was first collected from multiple public databases. Target annotations for 265 compounds were obtained from DrugBank (https://go.drugbank.com/; version 5.1.14; accessed 4 January 2026) and PubChem (https://pubchem.ncbi.nlm.nih.gov/; accessed on 5 January 2026) and linked to drug sensitivity data from the Genomics of Drug Sensitivity in Cancer (GDSC) database project (https://www.cancerrxgene.org/; Release 8.4; accessed 5 January 2026) [37], while 1574 naturally occurring anticancer compounds were collected from the Naturally Occurring Plant-based Anti-cancer Compound-Activity-Target database (NPACT; https://crdd.osdd.net/raghava/npact/; accessed on 15 January 2026), and their target information was retrieved from the Traditional Chinese Medicine Systems Pharmacology Database and Analysis Platform (TCMSP; https://tcmsp-e.com/tcmsp.php; accessed on 12 January 2026), DrugBank, and PubChem [38,39,40,41]. Target names were standardized to approved HGNC gene symbols. Synonyms and protein names were mapped to their corresponding official gene symbols, and entries mapping to the same official symbol were merged. Records without an unambiguous human target annotation were excluded. After integration and deduplication, 1093 unique targets were retained and encoded as binary target vectors.

The previously published DeepMDS model was then used to evaluate the antitumor potential of candidate target combinations [21]. DeepMDS is a fully connected feedforward neural network regressor. Its 1308-dimensional input comprises 215 normalized gene expression features and a 1093-element multi-hot target vector. The published architecture contains two dense hidden layers with 200 and 100 units, respectively; each uses a rectified linear unit (ReLU) activation followed by dropout at a rate of 0.5. The output layer contains one linear unit that returns a pseudo-IC_50_ score. Model fitting in the original study used mean squared error loss, the Adam optimizer, a learning rate of 1 × 10^−5^, a batch size of 128, and 200 epochs. Hyperparameters were selected by grid search with 10-fold cross-validation, and the original implementation used Python 3.6 with Keras/Theano [21]. No new network architecture was developed in the present study. Lower pseudo-IC_50_ values indicate stronger predicted synergistic antitumor effects.

In this study, after integration and deduplication of the source databases, 1093 unique targets were retained. All unordered combinations without target repetition were generated: 596,778 two-target combinations and 217,028,266 three-target combinations, resulting in a total of 217,625,044 candidate target combinations. Each target set was encoded as a 1093-dimensional binary multi-hot vector, in which a value of 1 indicated inclusion of a target in the candidate combination and a value of 0 indicated its absence. This target vector was concatenated with the 215-dimensional gene-expression representation of the corresponding cell line, resulting in a 1308-dimensional input vector.

Based on the constructed target feature space, candidate two-target and three-target combinations were generated. These combination features were integrated with the gene expression profiles of MCF-7 and MDA-MB-231 cells to construct feature matrices. These feature matrices were then submitted to the DeepMDS model for prediction of pseudo-IC_50_ values. The predicted pseudo-IC_50_ values were subsequently used to rank candidate target combinations, and combinations with lower values were considered to possess stronger potential synergistic anti-breast cancer activity and were prioritized for further analysis and experimental validation.

### 4.3. Cell Culture

Human ER-positive luminal-type breast cancer cells MCF-7 (Cat. No. SCSP-531), triple-negative breast cancer cells MDA-MB-231 (Cat. No. SCSP-5043) and mouse breast cancer 4T1 (Cat. No. SCSP-5056) cell line were purchased from the Cell Bank of the Chinese Academy of Sciences (Shanghai, China). MCF-7 and MDA-MB-231 were cultured in DMEM medium supplemented with 10% FBS and 1% penicillin/streptomycin and incubated at 37 °C and 5% CO_2_. The 4T1 cells were cultured in RPMI-1640 medium supplemented with 10% FBS and 1% penicillin/streptomycin and incubated at 37 °C and 5% CO_2_.

### 4.4. Small Interfering RNA Design and Transfection

Small interfering RNAs (siRNAs) targeting *BIRC5*, *NAMPT*, *TOP1*, *HSP72*, and *MTOR* were designed using the DSIR tool (https://biodev.cea.fr/DSIR/DSIR.html; accessed on 25 February 2026) against the RefSeq transcripts listed in Table 4 and were synthesized by GenePharma (Suzhou, China). A sequence-scrambled siRNA with no known target in the human transcriptome was used as the negative-control siRNA (NC). All siRNAs were dissolved in RNase-free water to prepare 20 μM stock solutions.

MCF-7 and MDA-MB-231 cells were transfected using GP-transfect-Mate according to the manufacturer’s instructions. For each three-gene candidate set, the siRNAs were evaluated individually, in all three pairwise combinations, and as a triple combination. To minimize potential confounding caused by differences in the total oligonucleotide load, the single- and pairwise-siRNA groups were supplemented with NC siRNA so that the total amount of siRNA was identical across all transfected groups. The same amount of GP-transfect-Mate was used in all transfected groups. Blank-control cells (BC) received neither siRNA nor transfection reagent.

After incubation with the siRNA-transfection reagent complexes for 5 h at 37 °C, the transfection medium was removed and replaced with fresh complete growth medium. Cells were subsequently cultured for 48 h before RT-qPCR analysis or the CCK-8 assay. The siRNA sequences are listed in Table 4.

### 4.5. RT-qPCR

MCF-7 and MDA-MB-231 cells were seeded in 6-well plates at a density of 1.5 × 10^5^ cells/well and cultured overnight. Transfection was performed when cells reached approximately 60% confluence.

For each well, 6 μL of the corresponding 20 μM target-siRNA stock solution was used for each target. The total siRNA volume was maintained at 18 μL per well by supplementation with NC siRNA. Accordingly, a single-siRNA group received 6 μL of target siRNA and 12 μL of NC siRNA; a triple-siRNA group received 6 μL of each of the three target siRNAs; and the NC group received 18 μL of NC siRNA. The siRNA solution was diluted in 200 μL of serum-free medium. Separately, 10 μL of GP-transfect-Mate was diluted in another 200 μL of serum-free medium. The two solutions were combined, gently mixed, and incubated at room temperature for 20 min to allow formation of siRNA-reagent complexes. The resulting complexes were added to each well containing 1.6 mL of culture medium. After incubation for 5 h at 37 °C, the transfection medium was removed, the cells were washed once with PBS, and fresh complete growth medium was added. Cells were cultured for a further 48 h before RNA isolation.

Cells were washed once with PBS, and total RNA was extracted using the RNA-Quick Purification Kit (YiShan Bio-tech) according to the manufacturer’s instructions. Complementary DNA was synthesized using the Fast All-in-One RT Kit (YiShan Bio-tech). Quantitative real-time PCR was performed on an ABI 7500 Real-Time PCR System (Applied Biosystems, Foster City, CA, USA) using the qPCR Master Mix (YiShan Bio-tech) according to the manufacturer’s protocol. Relative gene expression was normalized to *GAPDH* and calculated using the 2^−ΔΔCt^ method, with the BC group used as the calibrator. The primer sequences are listed in Table 5. Three replicate wells were included for each condition. Notably, the primer pair originally designated as targeting HSP72 anneals to a conserved region shared by the human HSPA1A and HSPA1B transcripts and is predicted to generate the same amplicon from both transcripts. Post-amplification dissociation-curve analysis was performed using the original instrument data. The corresponding derivative melting curves are provided in Supplementary Figure S1. It was therefore used as a pan-HSPA1A/HSPA1B assay to determine their combined transcript abundance rather than the expression of either transcript individually.

### 4.6. In Vitro Anticancer Effects of Single and Combined siRNA Transfection

To determine the individual and combined contributions of the predicted targets, a complete set of single-, pairwise-, and triple-siRNA conditions was evaluated for each candidate three-gene combination. For the BIRC5-NAMPT-TOP1 candidate set, the treatment groups were BIRC5, NAMPT, TOP1, BIRC5 + NAMPT, BIRC5 + TOP1, NAMPT + TOP1, and BIRC5 + NAMPT + TOP1 siRNAs. For the HSP72-NAMPT-MTOR candidate set, the corresponding groups were HSP72, NAMPT, MTOR, HSP72 + NAMPT, HSP72 + MTOR, NAMPT + MTOR, and HSP72 + NAMPT + MTOR siRNAs. An NC group transfected with non-targeting siRNA and a BC group receiving neither siRNA nor transfection reagent were included.

MCF-7 and MDA-MB-231 cells were seeded in 96-well plates at densities of 5 × 10^3^ and 1 × 10^4^ cells per well, respectively, and cultured for 24 h at 37 °C in a humidified atmosphere containing 5% CO_2_. For each target siRNA, 0.3 μL of the 20 μM siRNA stock solution was used. The total siRNA volume was maintained at 0.9 μL per well by supplementation with NC siRNA. Thus, a single-siRNA group received 0.3 μL of target siRNA and 0.6 μL of NC siRNA; a pairwise-siRNA group received 0.3 μL of each of the two target siRNAs and 0.3 μL of NC siRNA; a triple-siRNA group received 0.3 μL of each of the three target siRNAs; and the NC group received 0.9 μL of NC siRNA.

The siRNA solution was diluted in 10 μL of serum-free medium. Separately, 0.8 μL of GP-transfect-Mate was diluted in another 10 μL of serum-free medium. The two solutions were combined and incubated at room temperature for 20 min. The resulting siRNA-reagent complexes were added to wells containing 80 μL of culture medium. After incubation for 5 h at 37 °C, the transfection medium was removed, the cells were washed once with PBS, and fresh complete growth medium was added. Cells were then cultured for a further 48 h.

Cell viability was evaluated using the Cell Counting Kit-8 (CCK-8). Briefly, the culture medium was replaced with 90 μL of fresh medium containing 10 μL of CCK-8 solution, and cells were incubated for 1–2 h at 37 °C. Absorbance was measured at 450 nm using a Synergy H1 microplate reader (BioTek, Winooski, VT, USA). Cell-free wells containing culture medium and CCK-8 solution were used for background subtraction. Cell viability was calculated as follows:

(1) $$cell\;viability\left(\%\right)=\frac{A_{sample}-A_{background}}{A_{BC}-A_{background}}\times 100\%$$

where *A*_sample_ was the absorbance of cells after treatment, A_BC_ was the absorbance of cells without transfection, and *A*_background_ was the absorbance of blank well without cells.

### 4.7. In Vitro Anticancer Activity of Corresponding Inhibitor Combination

To pharmacologically translate the synergistic target combination identified at the gene level, BIRC5, NAMPT, and TOP1 were matched with their corresponding small-molecule inhibitors, LQZ-7I, FK866, and topotecan, respectively [33,42,43]. The anticancer effects of these inhibitors, either alone or in combination, were evaluated in MCF-7, MDA-MB-231, and 4T1 cells using the CCK-8 assay.

The following experimental groups were established: a control group, LQZ-7I monotherapy group, FK866 monotherapy group, topotecan monotherapy group, and a combination therapy group (LFT): LQZ-7I + FK866 + topotecan. LQZ-7I and topotecan were dissolved in DMSO, while FK866 was dissolved in absolute ethanol. Stock solutions were diluted with the corresponding culture medium immediately before treatment. MCF-7 and MDA-MB-231 cells were treated in DMEM-based medium, whereas 4T1 cells were treated in RPMI-1640-based medium.

For single-agent concentration–response experiments, LQZ-7I was evaluated at 0.01, 0.1, 1, 10, and 100 μM, and topotecan was evaluated at 0.001, 0.01, 0.1, 1, and 10 μM. FK866 was evaluated at 50, 100, 150, 200, and 300 μM in MCF-7 cells and at 1, 10, 100, 200, and 300 μM in MDA-MB-231 and 4T1 cells. In preliminary 48 h CCK-8 experiments, lower FK866 concentrations did not produce a dose-effect response spanning the 50% viability level under the present experimental conditions. The FK866 concentration range was therefore extended empirically to a maximum of 300 μM to encompass the median-effect region and permit estimation of an apparent 48 h CCK-8-derived IC_50_. This upper concentration was used solely for in vitro dose–response characterization and was not intended to represent a clinically relevant exposure or the intrinsic biochemical potency of FK866 toward NAMPT.

For combination experiments, LQZ-7I, FK866, and topotecan were evaluated at four predefined fixed molar ratios of 2.5:10:1, 2.5:0.1:1, 10:10:1, and 10:0.1:1. At each concentration level, the concentrations of all three components changed proportionally while maintaining the designated molar ratio. The total nominal mixture concentrations were 0.1, 0.5, 1, 2.5, 5, and 10 μM for MCF-7 cells and 0.001, 0.01, 0.1, 1, 10, and 50 μM for MDA-MB-231 and 4T1 cells.

MCF-7, MDA-MB-231, and 4T1 cells were seeded in 96-well plates at densities of 5 × 10^3^, 1 × 10^4^, and 5 × 10^3^ cells per well, respectively. Cells were exposed to the individual compounds or combinations for 48 h, after which CCK-8-derived cell viability was determined. Apparent IC_50_ values were estimated from the concentration-response data. Drug interactions were evaluated using CompuSyn software (version 1.0) according to the Chou-Talalay method [44]. The combination index (CI) values were calculated by CompuSyn from the complete single-agent and fixed-ratio dose-effect datasets and the CI values reported in Table 2 correspond to *F*a = 0.50. Here, CI > 1 indicates antagonism, CI = 1 indicates additive effects, and CI < 1 indicates synergy. For the three-drug combination, the CI at the 50% effect level was expressed as (Equation (2)):

(2) $$CI=\frac{D_{A}}{{IC}_{x,A}}+\frac{D_{B}}{{IC}_{x,B}}+\;\frac{D_{C}}{IC_{x,C}}$$

where *D_A_*, *D_B_*, and *D_C_* represent the concentration of LQZ-7I, FK866, and topotecan, respectively, within the combination producing the required 50% effect, whereas *IC_x_*_,*A*_, *IC_x_*_,*B*_, and *IC_x_*_,*C*_ represent the corresponding single-agent concentrations producing the same effect.

### 4.8. In Vivo Evaluation of Antitumor Efficacy and Safety

#### 4.8.1. Establishment of the Breast Cancer Model and Experiment Design

Specific-pathogen-free (SPF) female BALB/c mice (5–6 weeks old, weighing 18–20 g) were obtained from Cavens Laboratory Animal Co., Ltd. (Changzhou, China). Mice were housed in SPF-grade conditions at the Laboratory Animal Center of Jiangsu University. They had continuous access to drinking water, feed, and bedding materials, and were kept in a controlled environment with a temperature of 20–25 °C, humidity of 45–55%, and clean air. The experimental protocol was reviewed and approved by the Animal Ethics Committee of Jiangsu University.

To establish the tumor-bearing mouse model, 0.15 mL of 4T1 cell suspension (2 × 10^7^ cells/mL) was inoculated subcutaneously into the left axilla of each BALB/c mouse. Once the tumor volume reached approximately 50–100 mm^3^, mice were randomly divided into eight groups (*n* = 5 per group; Table 6) and treated via intraperitoneal injection. Drugs or corresponding vehicle preparations were administered intraperitoneally at a dosage of 0.1 mL per 10 g of body weight.

The initially planned administration interval was every 2 days. Accordingly, all groups received the assigned drug or corresponding vehicle on Days 0, 2, 4, 6, and 8. At the Day 8 assessment, body-weight loss, reduced activity, and deterioration in general condition were observed in the FK866 + topotecan and LQZ-7I + FK866 + topotecan groups. Following the Day 8 administration and welfare assessment, the dosing interval was extended from 2 to 6 days for all eight groups. The next and final administration was performed on Day 14. The study endpoint was Day 16.

Stock solutions of each drug were prepared as follows: LQZ-7I was dissolved sequentially in DMSO, polyoxyethylene castor oil, and 20% sodium sulfobutylether-β-cyclodextrin saline to a final concentration of 0.38 mg/mL. FK866 was dissolved in propylene glycol, Tween 80, and pure water to a final concentration of 1.7 mg/mL. Topotecan was dissolved in saline to a concentration of 0.2 mg/mL. Blank control mice received corresponding vehicle solutions. All preparations were freshly made before each injection.

#### 4.8.2. Monitoring of Tumor Growth and Humane Endpoints

Mice were monitored daily for changes in body weight, fur texture, behavior, and overall health status. Tumor length and width were measured using a digital caliper on Days 0, 2, 4, 6, 8, 12, 14, and 16. Tumor volume was calculated according to Equation (3). Body weight and tumor growth curves were plotted based on the mice’s body weight and tumor volume.

(3) $$V=\frac{\pi }{6}\times length\times {width}^{2}$$

According to the animal protocol approved by the Animal Ethics Committee of Jiangsu University, tumor volume in the subcutaneous tumor model was generally required not to exceed 2000 mm^3^. Animals reaching this tumor-burden criterion or showing severe deterioration in general condition were to be removed from the study and euthanized according to the approved protocol.

On Day 16, final tumor measurements were performed. Mice were then anesthetized, and terminal blood samples were collected into EDTA-treated tubes for hematological analysis and non-anticoagulant tubes for serum-biochemistry analysis. Following blood collection, mice were euthanized according to the approved protocol. Tumors were excised, photographed, weighed, and fixed in 4% paraformaldehyde. The tumor-inhibition rate was calculated from the mean terminal tumor weight of each group according to Equation (4).

(4) $$Tumor\;inhibition\;rate=\frac{Tumor\;weight\;in\;the\;control\;group-Tumor\;weight\;in\;experimental\;group}{Tumor\;weight\;in\;the\;control\;group}\times 100\%$$

#### 4.8.3. Determination of Liver and Kidney Function

Blood samples without anticoagulant were allowed to clot for 30 min at room temperature and centrifuged at 4000 rpm for 10 min to isolate serum. According to the instructions of the assay kits, the serum levels of aspartate aminotransferase (AST; Liver Function (AST + ALT) Assay Kit Set, Cat. No. BC-S001), alanine aminotransferase (ALT; Liver Function (AST + ALT) Assay Kit Set, Cat. No. BC-S001), total bilirubin (TBIL; Cat. No. BC6465), creatinine (CRE; Cat. No. BC683F), and blood urea nitrogen (BUN; Cat. No. BC1535) were measured using corresponding assay kits purchased from Beijing Solarbio Science & Technology Co., Ltd. (Beijing, China) according to the manufacturer’s protocols.

#### 4.8.4. Terminal Hematological Analysis

Whole blood collected in EDTA-treated tubes on Day 16 was gently mixed and stored upright at 4 °C. Hematological analysis was performed within 24 h using an automated blood analyzer (ADVIA 2120i, Bayer, Germany). Parameters measured included white blood cell count (WBC), lymphocyte count (Lymph#), monocyte count (Mon#), neutrophil count (Gran#), lymphocyte percentage (Lymph%), monocyte percentage (Mon%), neutrophil percentage (Gran%), red blood cell count (RBC), hemoglobin (HGB), hematocrit (HCT), mean corpuscular volume (MCV), mean corpuscular hemoglobin (MCH), mean corpuscular hemoglobin concentration (MCHC), red cell distribution width coefficient of variation (RDW), platelet count (PLT), mean platelet volume (MPV), platelet distribution width (PDW), and plateletcrit (PCT).

### 4.9. Statistical Analysis

All data were expressed as mean ± standard deviation (SD) and analyzed using SPSS Statistics version 22.0. The difference between two groups was analyzed using Student’s *t*-test, whereas comparisons among multiple groups were performed using one-way analysis of variance (ANOVA). A *p*-value of less than 0.05 was considered statistically significant. The experimental results were visualized using GraphPad Prism 8.0.

## 5. Conclusions

In summary, this study applied the previously developed DeepMDS model for prioritizing higher-order synergistic therapeutic target combinations for breast cancer. Among the predicted candidates, the combination of BIRC5, NAMPT, and TOP1 received the top relative rank in both luminal-like (MCF-7) and triple-negative (MDA-MB-231) breast cancer cell lines and produced the greatest reduction in cell viability among the target sets examined. The corresponding inhibitor combination, including LQZ-7I, FK866, and topotecan, showed empirical pharmacological interactions at several fixed ratios and reduced tumor burden in a schedule-adapted 4T1 experiment. These findings identify BIRC5-NAMPT-TOP1 as a candidate higher-order target combination for further investigation. These findings not only offer a promising multi-targeted therapeutic strategy for breast cancer but also validate the utility of deep learning-based synergy prediction in accelerating inhibitor combination discovery. The approach demonstrated in this study may be extended to other cancer types and molecular subtypes, providing a valuable framework for rational drug development and personalized combination therapies in oncology.

## Figures and Tables

**Table 1:** Predicted pseudo-IC_50_ values for target combinations in MCF-7 and MDA-MB-231 cells.

Target 1	Target 2	Target 3	MCF-7		MDA-MB-231	
			Predicted Values	Rank	Predicted Values	Rank
BIRC5	NAMPT	TOP1	−13.23	1	−15.09	1
BIRC5	TOP1	CDK7	−12.64	3	−14.04	2
BIRC5	HSP72	TOP1	−13.04	2	−14.00	3
HSP72	NAMPT	TOP1	−11.53	11	−13.99	4
HSP72	NAMPT	MTOR	−8.61	850	−11.70	37
TOP1	PLK1	ESR1	−7.50	2871	−8.26	5200
PI3Kα	TOP1	ESR1	−6.57	7855	−6.82	15,403

Pseudo-IC_50_ values are relative model outputs used for candidate prioritization and are not experimentally measured IC_50_ values or direct evidence of biological synergy. Lower values indicate higher model-based prioritization.

**Table 2:** Effects of LQZ-7I, FK866, topotecan and their combination on cell viability.

Group	MCF-7		MDA-MB-231		4T1	
	IC_50_ (μM)	CI	IC_50_ (μM)	CI	IC_50_ (μM)	CI
LQZ-7I	6.75	-	79.85	-	41.38	-
FK866	253.5	-	93.85	-	91.01	-
Topotecan	2.8	-	9.09	-	6.76	-
LFT (2.5:10:1)	7.04	0.26	13.16	0.19	10.35	0.15
LFT (2.5:0.1:1)	2.74	0.43	5.15	0.21	8.57	0.36
LFT (10:10:1)	11.46	0.82	17.8	0.23	23.05	0.42
LFT (10:0.1:1)	4.32	0.35	7.79	0.37	8.77	0.65

CI, combination index at the 50% effect level; IC_50_, half-maximal inhibitory concentration; -, not applicable; LFT, combination of LQZ-7I, FK866, and topotecan. For the combination groups, IC_50_ represents the total nominal concentration of the three-drug mixture rather than the concentration of any individual component. The concentration of each component changed proportionally according to the indicated molar ratio.

**Table 3:** Effects of LQZ-7I, FK866, topotecan and their combinations on tumor inhibition rate.

Group	Tumor Weight (g)	Tumor Inhibition Rate (%)
LQZ-7I	1.32 ± 0.21	20.48
FK866	1.53 ± 0.26	7.83
Topotecan	1.26 ± 0.58	24.10
LQZ-7I + FK866	1.07 ± 0.42	35.54
LQZ-7I + Topotecan	0.78 ± 0.20	53.01
FK866 + Topotecan	0.84 ± 0.22	49.40
LQZ-7I + FK866 + Topotecan	0.63 ± 0.15	62.05
Control	1.66 ± 0.54	-

**Table 4:** Sequences of the siRNAs used for target-gene silencing.

Gene	RefSeq Accession No.	Sense (5′-3′)	Antisense (5′-3′)
*BIRC5*	NM_001168.3	GAAGCAGUUUGAAGAAUUATT	UAAUUCUUCAAACUGCUUCTT
*NAMPT*	NM_005746.3	CCACCCAACACAAGCAAAGUUUAUU	AAUAAACUUUGCUUGUGUUGGGUGG
*TOP1*	NM_003286.4	GGGAAGGACUCCAUCAGAUACUAUA	UAUAGUAUCUGAUGGAGUCCUUCCC
*HSPA1A*/*HSPA1B* (*HSP72*) ^a^	NM_005345.6; NM_005346.6	AGGACGAGUUUGAGCACAATT	UUGUGCUCAAACUCGUCCUTT
*MTOR*	NM_004958.4	GUAAAUGCUUCCACUAAACTT	GUUUAGUGGAAGCAUUUACTT
Negative		UUCUCCGAACGUGUCACGUTT	ACGUGACACGUUCGGAGAATT

^a^ The targeting sequence of this siRNA, excluding the terminal dTdT overhang, is fully conserved in the human HSPA1A (NM_005345.6) and HSPA1B (NM_005346.6) transcripts. The siRNA is therefore expected to target both transcripts and is referred to herein as the HSPA1A/HSPA1B-targeting siRNA or HSP72 siRNA.

**Table 5:** Primer sequences used for RT-qPCR analysis.

Gene	Forward (5′-3′)	Reverse (5′-3′)
*BIRC5*	CCGCATCTCTACATTCAAGAAC	CTCCTTGAAGCAGAAGAAACAC
*NAMPT*	AGCAGAACACAGTACCATAACA	CCCATATTTTCTCACACGCATT
*TOP1*	GTTCTTTGCAAAAATGCTCGAC	GAAATACTGGCTCATCTGGGTA
*HSPA1A*/*HSPA1B* (pan-HSP72) ^a^	CCTGGAGTCCTACGCCTTCAAC	TGTCCGCCTCGCTGATCTTG
*M* *TOR*	GAGATACGCTGTCATCCCTTTA	CTGTATTATTGACGGCATGCTC
*GAPDH*	CAGGAGGCATTGCTGATGAT	GAAGGCTGGGGCTCATTT

^a^ The primer pair originally designated as targeting *HSP72* anneals to a conserved region shared by the human *HSPA1A* and *HSPA1B* transcripts. It was therefore used as a pan-*HSPA1A*/*HSPA1B* assay to determine their combined transcript abundance rather than the expression of either transcript individually.

**Table 6:** Experimental grouping and dosages (*n* = 5).

Group	LQZ-7I (mg/kg)	FK866 (mg/kg)	Topotecan (mg/kg)
LQZ-7I	3.8	-	-
FK866	-	17	-
Topotecan	-	-	2
LQZ-7I + FK866	3.8	17	-
LQZ-7I + Topotecan	3.8	-	2
FK866 + Topotecan	-	17	2
LQZ-7I + FK866 + Topotecan	3.8	17	2
Control	-	-	-

## Data Availability

The original contributions presented in this study are included in the article and the Supplementary Materials. Further inquiries can be directed to the corresponding author.

## Supplementary Materials

The following supporting information can be downloaded at: https://www.mdpi.com/article/10.3390/ph19091363/s1. Supplementary Figure S1. Melting-curve analysis of the pan-HSPA1A/HSPA1B RT-qPCR assay. Derivative melting curves obtained from the original post-amplification dissociation data for MCF-7 (A) and MDA-MB-231 (B) samples. The pan-HSPA1A/HSPA1B assay reactions showed a single dominant melting peak at approximately 84 °C, with closely overlapping profiles and no evident secondary peaks or shoulders over the recorded temperature range. These results support the presence of a single predominant amplification product under the applied RT-qPCR conditions. Because the primer pair recognizes a conserved region shared by HSPA1A and HSPA1B and is predicted to generate the same amplicon from both transcripts, the melting-curve analysis supports product homogeneity but does not distinguish the two paralogous transcripts. Supplementary Figure S2. ShinyTHOR-based descriptive visualization of the investigated target genes across breast-derived cancer cell lines. The Multiple Analytes module of ShinyTHOR was used to visualize the basal expression patterns of HSPA1A, HSPA1B, NAMPT, BIRC5, TOP1, and MTOR. The following filters were applied: Site, breast; Histology, All; and Pathology, All. The heatmap displays the scaled-expression values generated by ShinyTHOR, with darker blue indicating higher displayed expression values. Genes and cell lines were hierarchically clustered by the platform. The upper annotation bars indicate the pathology and histology metadata associated with each cell line. MCF7, corresponding to MCF-7, and MDA-MB-231 are outlined for ease of identification. The visualization was used descriptively to assess inter-cell-line expression heterogeneity and was not treated as a formal differential-expression or target-dependency analysis.

## Abbreviations

The following abbreviations are used in this manuscript:

ALT	alanine aminotransferase
ANOVA	analysis of variance
AST	aspartate aminotransferase
BC	blank control
BIRC5	baculoviral IAP repeat containing 5
BUN	blood urea nitrogen
CCK-8	Cell Counting Kit-8
CDK7	cyclin-dependent kinase 7
CI	combination index
CRE	creatinine
DMEM	Dulbecco’s modified Eagle medium
DMSO	dimethyl sulfoxide
DNA	deoxyribonucleic acid
EDTA	ethylenediaminetetraacetic acid
ESR1	estrogen receptor 1
FBS	fetal bovine serum
GAPDH	glyceraldehyde-3-phosphate dehydrogenase
GDSC	Genomics of Drug Sensitivity in Cancer
Gran#	granulocyte count
Gran%	granulocyte percentage
HCT	hematocrit
HER2	human epidermal growth factor receptor 2
HGB	hemoglobin
HSP72	heat shock protein 72
IC_50_	half-maximal inhibitory concentration
Lymph#	lymphocyte count
Lymph%	lymphocyte percentage
MCH	mean corpuscular hemoglobin
MCHC	mean corpuscular hemoglobin concentration
MCV	mean corpuscular volume
Mon#	monocyte count
Mon%	monocyte percentage
MPV	mean platelet volume
mRNA	messenger RNA
MTOR/mTOR	mechanistic target of rapamycin kinase
NAD^+^	oxidized nicotinamide adenine dinucleotide
NAMPT	nicotinamide phosphoribosyltransferase
NC	negative control
NPACT	Naturally Occurring Plant-based Anti-cancer Compound-Activity-Target database
PBS	phosphate-buffered saline
PCT	plateletcrit
PDW	platelet distribution width
PI3Kα	phosphatidylinositol 3-kinase alpha
PLK1	polo-like kinase 1
PLT	platelet count
RBC	red blood cell count
RDW-CV	red cell distribution width–coefficient of variation
RNA	ribonucleic acid
RPMI-1640	Roswell Park Memorial Institute 1640 medium
RT-qPCR	reverse-transcription quantitative polymerase chain reaction
SD	standard deviation
siRNA	small interfering RNA
SPF	specific-pathogen-free
TBIL	total bilirubin
TCMSP	Traditional Chinese Medicine Systems Pharmacology Database and Analysis Platform
TOP1	DNA topoisomerase I
WBC	white blood cell count
