# Supplementary material for: Artificial Intelligence-Guided Prioritization and Experimental Evaluation of Synergistic Target Combinations for Breast Cancer Therapy
Source: Pharmaceuticals (Basel). 2026 Aug 28;19(9):1363. doi: 10.3390/ph19091363 (PMC13610653; doi:10.3390/ph19091363)
Supplement: Supplementary file 1 [file pharmaceuticals-19-01363-s001.zip › pharmaceuticals-4474144-supplementary.pdf]

Supplementary Material

Methods

ShinyTHOR-based descriptive expression analysis

To assess the expression heterogeneity of the investigated targets across a broader panel of breast-derived cancer cell lines, the Multiple Analytes module of ShinyTHOR was used. "Gene Expression" was selected as the analyte module, and BIRC5, NAMPT, TOP1, HSPA1A, HSPA1B, and MTOR were entered as the analytes of interest. Cell lines were filtered using the following settings: Site, breast; Histology, All; and Pathology, All.

The platform-generated scaled-expression heatmap and the associated hierarchical clustering of genes and cell lines were used to visualize inter-cell-line expression heterogeneity. The pathology and histology annotations provided by ShinyTHOR were retained. MCF7 and MDA-MB-231 were outlined in the exported figure solely to facilitate their identification; the expression scale, clustering order, and underlying data were not otherwise modified.

Figures

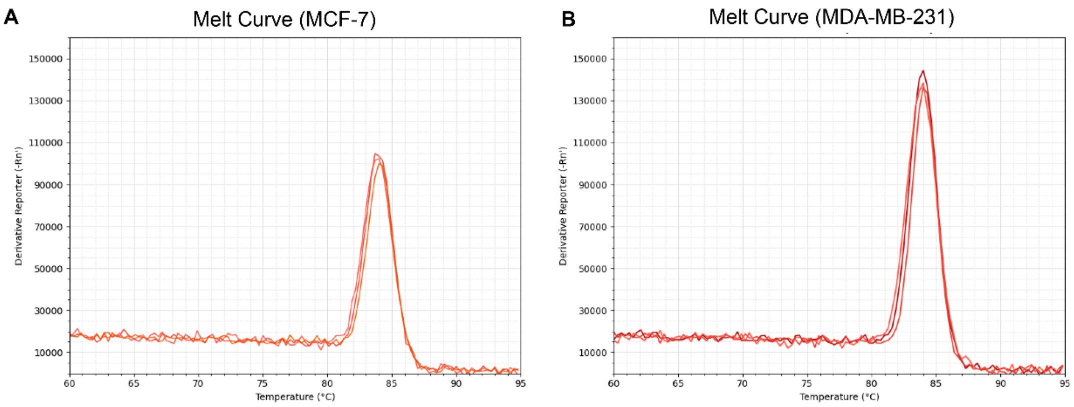

Supplementary Figure S1. Melting-curve analysis of the pan-HSPA1A/HSPA1B RT-qPCR assay. Derivative melting curves obtained from the original post-amplification dissociation data for MCF-7 (A) and MDA-MB-231 (B) samples. The pan-HSPA1A/HSPA1B assay reactions showed a single dominant melting peak at approximately 84 °C, with closely overlapping profiles and no evident secondary peaks or shoulders over the recorded temperature range. These results support the presence of a single predominant amplification product under the applied RT-qPCR conditions. Because the primer pair recognizes a conserved region shared by

HSPA1A and HSPA1B and is predicted to generate the same amplicon from both transcripts, the melting-curve analysis supports product homogeneity but does not distinguish the two paralogous transcripts.

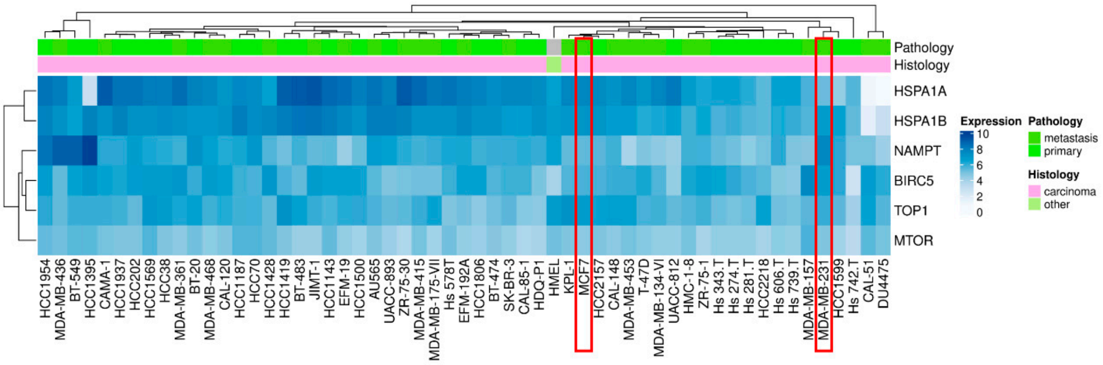

Supplementary Figure S2. ShinyTHOR-based descriptive visualization of the investigated target genes across breast-derived cancer cell lines. The Multiple Analytes module of ShinyTHOR was used to visualize the basal expression patterns of HSPA1A, HSPA1B, NAMPT, BIRC5, TOP1, and MTOR. The following filters were applied: Site, breast; Histology, All; and Pathology, All. The heatmap displays the scaled-expression values generated by ShinyTHOR, with darker blue indicating higher displayed expression values. Genes and cell lines were hierarchically clustered by the platform. The upper annotation bars indicate the pathology and histology metadata associated with each cell line. MCF7, corresponding to MCF-7, and MDA-MB-231 are outlined for ease of identification. The visualization was used descriptively to assess inter-cell-line expression heterogeneity and was not treated as a formal differential-expression or target-dependency analysis.
